# Autophagy degrades immunogenic endogenous retroelements induced by 5-azacytidine in acute myeloid leukemia

**DOI:** 10.1038/s41375-024-02250-6

**Published:** 2024-04-16

**Authors:** Nandita Noronha, Chantal Durette, Maxime Cahuzac, Bianca E Silva, Justine Courtois, Juliette Humeau, Allan Sauvat, Marie-Pierre Hardy, Krystel Vincent, Jean-Philippe Laverdure, Joël Lanoix, Frédéric Baron, Pierre Thibault, Claude Perreault, Gregory Ehx

**Affiliations:** 1 IRIC, Université de Montréal, Montreal, QC Canada; 2 GIGA Institute, Laboratory of Hematology, University of Liege, Liege, Belgium; 3 Equipe labellisée par la Ligue contre le Cancer, Université de Paris, Sorbonne Université, Inserm U1138, Institut Universitaire de France, Paris, France

**Keywords:** Tumour immunology, Antigen presentation, Cancer genomics, Acute myeloid leukaemia

## Abstract

The hypomethylating agent 5-azacytidine (AZA) is the first-line treatment for AML patients unfit for intensive chemotherapy. The effect of AZA results in part from T-cell cytotoxic responses against MHC-I-associated peptides (MAPs) deriving from hypermethylated genomic regions such as cancer-testis antigens (CTAs), or endogenous retroelements (EREs). However, evidence supporting higher ERE MAPs presentation after AZA treatment is lacking. Therefore, using proteogenomics, we examined the impact of AZA on the repertoire of MAPs and their source transcripts. AZA-treated AML upregulated both CTA and ERE transcripts, but only CTA MAPs were presented at greater levels. Upregulated ERE transcripts triggered innate immune responses against double-stranded RNAs but were degraded by autophagy, and not processed into MAPs. Autophagy resulted from the formation of protein aggregates caused by AZA-dependent inhibition of DNMT2. Autophagy inhibition had an additive effect with AZA on AML cell proliferation and survival, increased ERE levels, increased pro-inflammatory responses, and generated immunogenic tumor-specific ERE-derived MAPs. Finally, autophagy was associated with a lower abundance of CD8^+^ T-cell markers in AML patients expressing high levels of EREs. This work demonstrates that AZA-induced EREs are degraded by autophagy and shows that inhibiting autophagy can improve the immune recognition of AML blasts in treated patients.

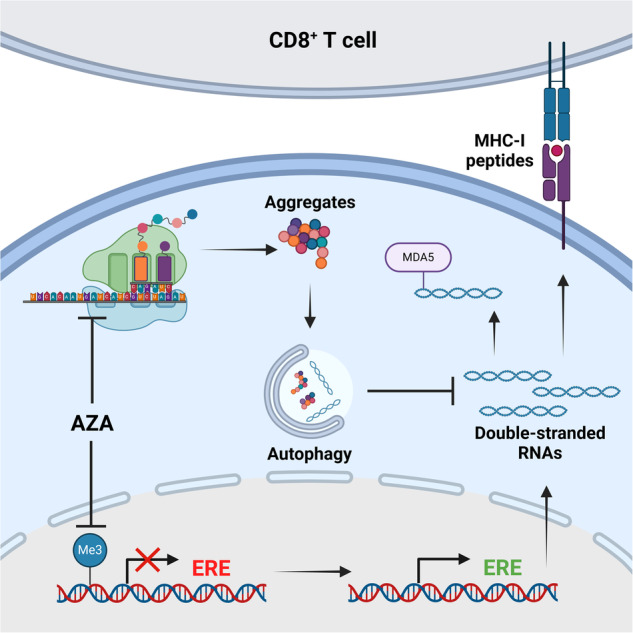

## Introduction

Acute myeloid leukemia (AML) is the most common acute leukemia in adults, with an overall 5-year survival below 30%. Standard therapy involves intensive chemotherapy with a ‘7 + 3’ regimen of cytarabine and anthracycline. Although AML is a heterogeneous disease, aberrant genomic methylation (hypermethylation in particular [1, 2]) is a hallmark of AML blasts. Therefore, hypomethylating agents such as 5-azacytidine (azacitidine, AZA) and 5-aza-2′-deoxycytidine (decitabine, DAC) are used as first-line therapy for AML patients unfit for intensive chemotherapy [3]. AZA is also used in maintenance therapy for fit patients without an *FLT3* mutation [3]. However, only 18–47% of patients respond to these therapies, stressing the need to improve therapy efficacy, possibly by combining them with other pharmacologic agents [4, 5].

AZA and DAC are cytidine nucleoside analogs that incorporate into genomic DNA during the mitosis [6]. High concentrations of AZA and DAC exert cytotoxic effects by inducing DNA double-strand breaks. However, at low concentrations, they act as suicide substrates for DNA methyltransferases (DNMTs) 1 and 3, leading to their degradation and the DNA demethylation of daughter cells. AZA differs from DAC by its ability to incorporate into RNA and DNA, thus inhibiting DNMT2, a transfer RNA methyltransferase [7]. While both agents have similar response rates in AML [8], only AZA significantly improves overall survival compared with conventional care regimens in phase III randomized trials [9, 10]. Therefore, only AZA is currently FDA-approved as a first-line treatment in AML [11].

In addition to their cytotoxic and demethylating effects, hypomethylating agents may mediate anti-leukemic activities by sustaining the elimination of malignant blasts by effector T cells [12]. Specifically, hypomethylating agents enhance the expression of transcripts coding for cancer-testis antigens [13, 14]. Cancer-testis antigens genes are normally silenced by genomic methylation and code for antigens deemed immunogenic because they are not expressed in normal MHC-positive somatic cells [15]. Accordingly, some studies evidenced that hypomethylating agents promote CD8^+^ T-cell activity [16], while others demonstrate a specific cytotoxic activity against cancer-testis antigens [14, 17, 18]. These studies suggest that hypomethylating agents-induced cancer-testis antigens can promote anti-leukemic CD8 T-cell reactions by generating immunogenic MHC-I-associated peptides (MAPs).

Along with cancer-testis antigens, hypomethylating agents promote the expression of endogenous retroelements (EREs) [19, 20]. EREs are highly repetitive sequences that are remnants of transposable elements incorporated into the human genome [21]. They can be segregated into LINEs and SINEs (long and short interspersed elements, respectively) and LTRs (long terminal repeats), the latter of which includes endogenous retroviruses (ERVs). EREs are epigenetically silenced mainly by genomic methylation in normal somatic cells [22], and dysregulated ERE expression is associated with several pathologic conditions, including autoimmunity, and cancer [23]. Hypomethylating agents-induced ERE overexpression in solid cancers leads to viral mimicry and concomitant innate immune response against ERE-derived double-stranded RNAs [24, 25]. Moreover, we and others have demonstrated that in addition to being expressed, EREs can be presented by MHC-I molecules and serve as immunogenic tumor antigens, notably in AML [26–29]. While EREs are perfect candidates for generating immunogenic MAPs following hypomethylating agents treatment, there is a lack of robust evidence to support that hypomethylating agents enhance their MAP presentation (and subsequent CD8^+^ T-cell responses) in AML.

Apart from the documented induction of cancer-testis MAPs and the assumed induction of ERE MAPs, the other impacts of hypomethylating agents on the immunopeptidome are poorly understood. Therefore, we sought to validate the induction of ERE MAPs by the most widely used hypomethylating agent, AZA, and to investigate its global effects on the immunopeptidome directly. We discovered that AZA promotes the expression of multiple cancer-testis MAPs but not ERE MAPs. Mechanistically, we demonstrate that the immunopeptidome of AZA-treated cells is shaped by autophagy, which degrades ERE transcripts and reduces their capacity to generate MAPs.

## Material and methods

All methods are reported in supplemental data.

## Results

### Low-dose AZA inhibits DNMT1 without inducing cell death

**Fig. 1:**
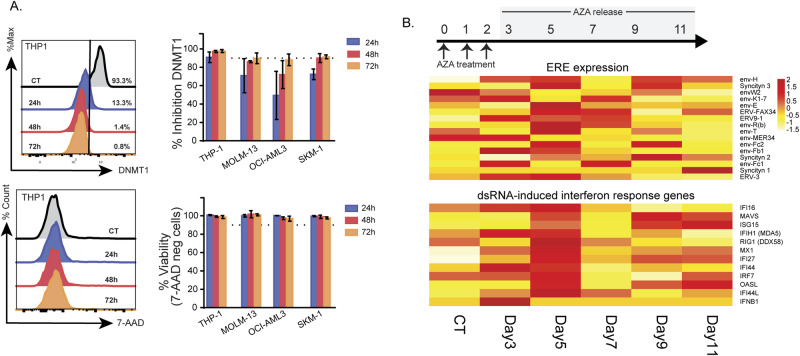
Low-dose AZA treatment leads to delayed, transient ERE and double-stranded RNA-induced interferon gene expression in AML cell lines. **A** Low AZA doses were added to four AML cell lines daily for three days (0.25 μM: MOLM-13 and SKM-1; 0.5 μM: THP-1 and OCI-AML3) and DNMT1 inhibition (upper panel) and cell viability were monitored by flow cytometry using 7-AAD (lower panel). The dotted lines represent 90% DNMT1 inhibition/viability in the upper and lower panels. The left panels depict representative histograms of THP-1 cells, while the right panels depict bar plots summarizing the percentage of expression/staining (as indicated in figures) of all four AML cells. Percentages were calculated by comparing AZA-treated cells to the control cells (CT). **B** Low AZA doses (0.5 μM) were added to THP-1 cells daily for three days, after which AZA treatment was released by washing and expanding cells in the absence of AZA. Cells were collected for RNA-seq at the time points highlighted in grey. Heatmap comparing RNA expression levels of EREs induced by AZA (upper panel) and genes involved in double-stranded RNA (dsRNA) -induced viral response (lower panel) identified in previous studies [24, 25] of control (day 3) and AZA-released cells at the time points indicated.

To investigate the effects of AZA on the immunopeptidome of AML, we selected four cell lines (THP-1, MOLM-13, SKM-1, and OCI-AML3) belonging to aggressive FAB types (M4/M5) and covering different but frequent mutational statuses (MLL-AF9, FLT3-ITD, TET2 (L1418fs), and NPM1c + DNMT3A (R882C), respectively). We aimed to focus on the hypomethylating effects of AZA (responsible for the induction of EREs). Therefore, we established a protocol enabling DNMT1 degradation and genomic demethylation without affecting viability or inducing DNA damage responses. AZA doses of 0.25 μM (for MOLM-13 and SKM-1) and 0.5 μM (for THP-1 and OCI-AML3) reduced cell growth, genome methylation, and DNMT1 expression without inducing cytotoxic effects (Fig. 1A and Fig. S1) and were chosen for further investigation.

Next, to maximize our chances of discovering ERE MAPs, we sought to identify a time point at which AZA induces an overexpression of ERE transcripts. Using THP-1 as a model, we performed RNA sequencing (RNA-seq) every 48 h from day 3 to 11 post-AZA discontinuation. We then quantified the expression of multiple ERE transcripts upregulated by AZA [25]. We also assessed the expression of genes involved in double-stranded RNA-induced interferon signaling in response to AZA-induced ERE expression [24] and observed that, together with ERE transcripts, they reached maximum expression around day 5 (72 h after the last AZA treatment; Fig. 1B). qPCR analyses further validated that the effect of AZA on ERE and double-stranded RNA-induced immune response genes was maximal on day 4 (48 h after the last AZA treatment; Fig. S2). Accordingly, we treated the four AML cell lines with these optimal AZA doses, administered three times at 24 h intervals (0 h, 24 h, and 48 h), and harvested the cells on day 4 to perform RNA-seq and mass spectrometry analyses, described in the next sections.

### AZA enhances the presentation of cancer-testis MAPs but not of ERE MAPs

**Fig. 2:**
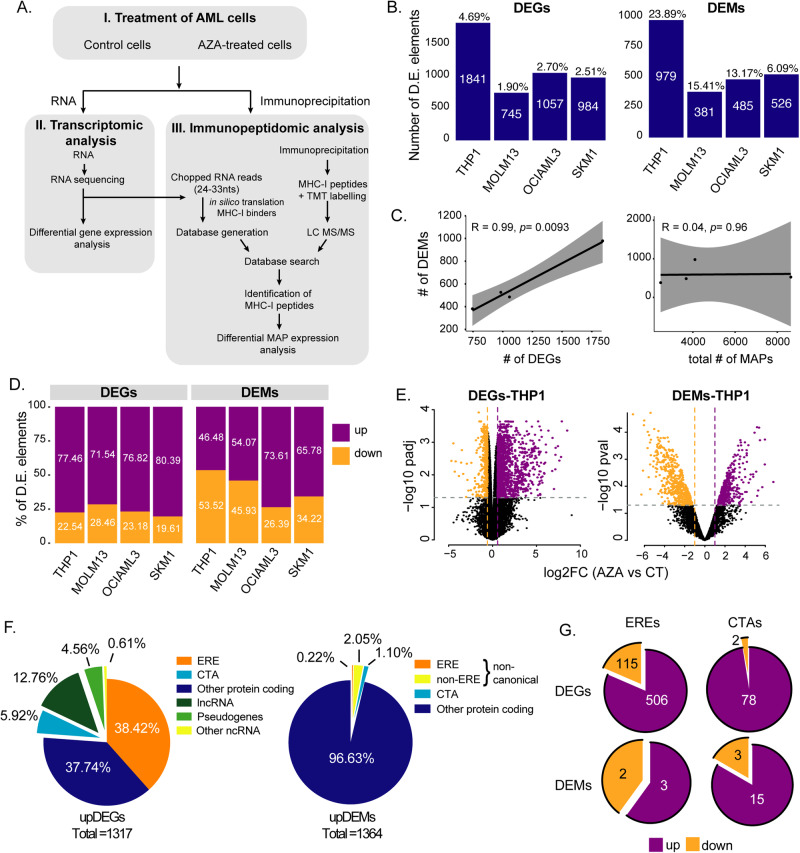
Proteogenomic characterization of AZA-mediated changes shows upregulation of MAPs derived from cancer-testis antigens but not from EREs. THP-1, MOLM-13, SKM-1, and OCI-AML3 cells were treated for 72 h with 0.5 µM of AZA (supplemented at 0, 24, and 48 h), washed, and maintained in fresh medium for 24 h before analysis. **A** Schematic representation of the study design for RNA-seq and mass spectrometry (MS) analyses. **B** The number of differentially expressed (D.E.) genes and MAPs (DEGs and DEMs, respectively) varies across cell lines. The numbers above the bars indicate the percentage of total genes or MAPs that were DEGs or DEMs, respectively. **C** Pearson correlation between the number of DEMs and DEGs (left panel) or DEMs and MAPs (right panel). Each dot corresponds to a cell line. **D** Percentage of D.E. elements up- or downregulated across cell lines. **E** Representative volcano plots of DEGs and DEMs between AZA (violet) and untreated (gold) THP-1 cells. **F** Pie charts depicting the percentage of biotypes of commonly upregulated transcripts (left) and MAPs (right) in all cell lines. The total number of upregulated D.E. elements is indicated below the pie charts. **G** Pie charts depicting the number of up- and downregulated EREs (left) and cancer-testis antigens (CTA, right) belonging to DEGs (upper panel) or DEMs (lower panel) fractions commonly regulated in all cell lines. lncRNA: long non-coding RNA, ncRNA: non-coding RNA.

To identify peptides, mass spectrometry relies on reference protein databases (such as Uniprot) to match each acquired spectrum to a peptide sequence. However, public databases do not contain non-exonic sequences, preventing the analysis of MAPs deriving from non-conventional transcripts, such as EREs. Therefore, we built personalized databases containing the MAP sequences corresponding to the RNA transcripts expressed by our cell lines in RNA-seq analyses (Fig. 2A and Fig. S3A, see methods for details). These databases enabled the identification of MAPs deriving from any expressed genomic region (including EREs). Following mass spectrometry identifications, differential abundance analyses were performed on each cell line to characterize the impact of AZA on the immunopeptidome. In parallel, differential gene expression analyses were conducted on RNA-seq data to evaluate the effects of AZA on the expression of coding genes and the ~4.2 × 10^6^ EREs reported in Repeatmasker [30, 31].

Altogether, these analyses revealed that the number of elements differentially expressed by AZA-treated cells varies across cell lines, with THP-1 being the most sensitive (Fig. 2B and Table S1). Overall, the proportion of differentially expressed MAPs (DEMs) was five times greater than that of differentially expressed transcripts (DEGs): 6–23% vs. 1.9–4.69%, respectively. Notably, the number of DEMs per cell line correlated almost perfectly with the number of DEGs rather than the total number of MAPs per cell line, showing that transcriptomic alterations are reflected in the immunopeptidome (Fig. 2C). However, the directionality of differential expression differed for MAPs and transcripts. While most DEGs ( > 70%) were upregulated by AZA, this was not the case for DEMs (Fig. 2D, E, Table S2). This means that, as with other drugs [32], changes in the immunopeptidome post-AZA treatment result from differential mRNA expression and post-translational events.

Regarding EREs, 506 were consistently upregulated in a unique differential gene expression analysis for each cell line (Fig. S3B). Using ERE distribution in the genome as a reference, we found that AZA selectively upregulated two classes of EREs, LINEs and LTRs, while SINEs were poorly upregulated (Fig. S3C). This was consistent with the fact that repression of SINEs depends mainly on histone methylation rather than DNA methylation [33]. While EREs represented a large proportion of DEGs ( ~ 38%), they accounted for only 0.22% of upregulated DEMs, suggesting that AZA-induced ERE transcripts may not be processed adequately for MHC-I presentation (Fig. 2F, G and Table S3). Because of the delay between AZA treatment and ERE induction (Fig. 1B), we repeated our immunopeptidomic analyses at a later time point (day 7) on THP-1 (the cell line with the highest AZA-induced EREs). We did not observe a higher ERE MAP presentation than on day 4 (Fig. S4). Notably, on both day 4 and day 7, upregulated DEMs contained more cancer-testis MAPs than ERE MAPs (Fig. 2F, G and Fig. S4). Among the upregulated DEMs, 152 ( ~ 10%) were AZA-specific (i.e., presented by all three AZA-treated replicates but undetected in control cells; Fig. S5A). MAPs induced de novo by AZA contained cancer-testis antigens but not ERE MAPs (Fig. S5B). We conclude that at the immunopeptidomic level, AZA upregulates the expression of cancer-testis MAPs (some being AZA-specific) but not ERE MAPs.

### AZA-induced EREs trigger innate immune responses

**Fig. 3:**
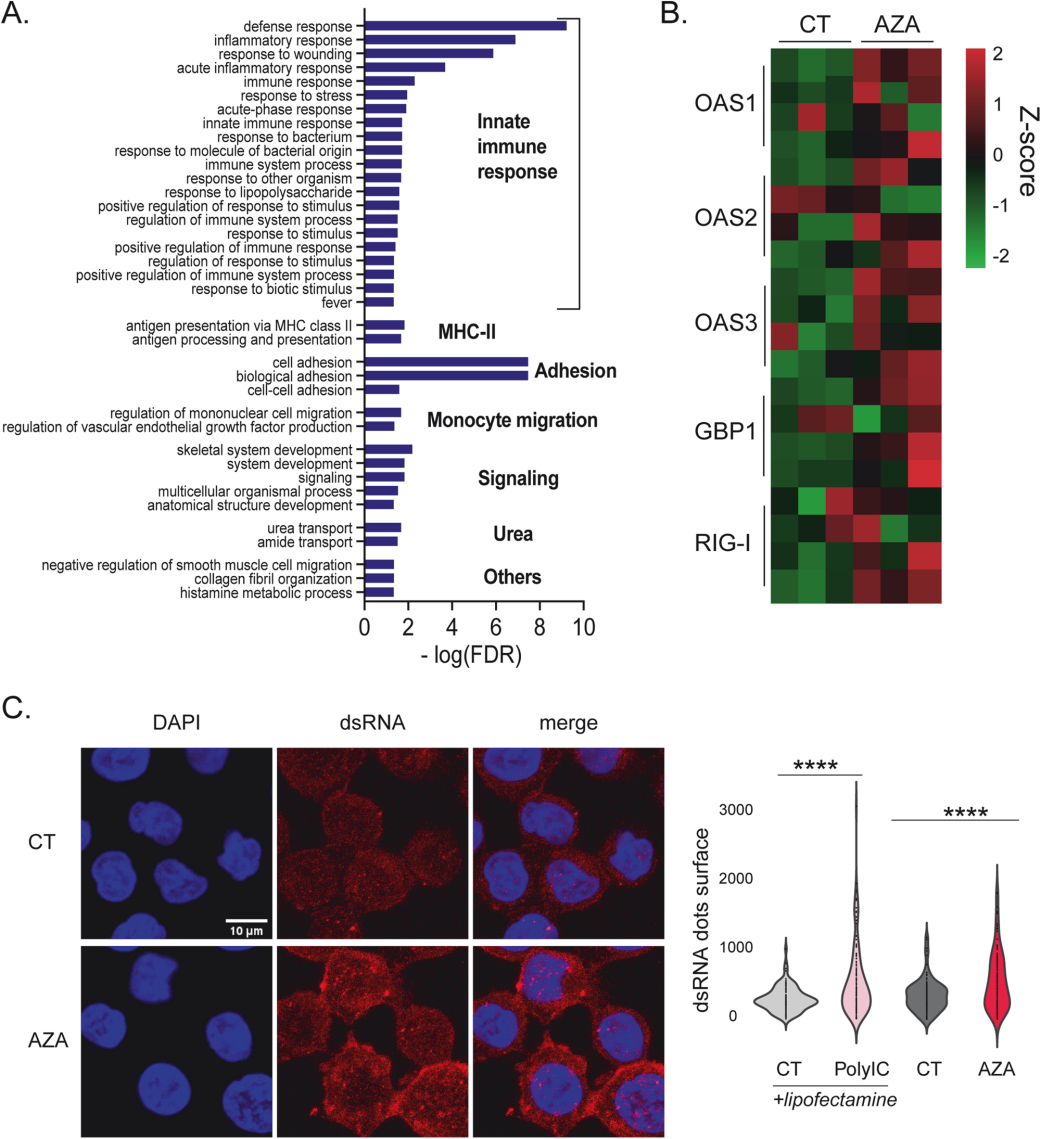
AZA-induced EREs trigger innate immune responses. **A** Histogram depicting GO-term analysis of the most significantly enriched biological processes associated with upregulated DEGs across all cell lines after AZA treatment. **B** Heatmap of genes involved in anti-double-stranded RNA (dsRNA) responses commonly regulated in all cell lines. **C** Representative images (left) and quantification (right) of dsRNA signals in THP-1 cells from microscopy images (two independent experiments). THP-1 cells transfected with 10 μg/ml polyinosinic:polycytidylic acid using lipofectamine (PolyIC+lipofectamine) were used as a positive control and were compared with cells treated with lipofectamine alone (CT+lipofectamine); (unpaired *t*-test; ****p < 0.0001).

In cancer cells, innate immune responses benefit the host because they can initiate cancer cell apoptosis and increase their adjuvanticity [34]. Because our analyses showed that the effects of AZA on EREs are much more conspicuous at the transcriptomic than at the immunopeptidomic level, we investigated whether AZA-induced ERE transcripts directly affect AML biology. We first performed a gene ontology (GO) analysis on the DEGs upregulated by AZA. This revealed that a large fraction of the identified pathways ( ~ 55%) were related to innate immune responses (Fig. 3A). Given that EREs can generate double-stranded RNAs recognized by anti-viral innate pathways, we examined the expression of multiple genes (OAS1, OAS2, OAS3, GBP1, and RIG-I) playing pivotal functions in such pathways. All of them were expressed at higher levels in AZA-treated cells (Fig. 3B). Accordingly, using microscopy, we observed greater amounts of double-stranded RNAs in AZA-treated cells (Fig. 3C). Altogether, these data show that the upregulation of ERE transcripts induced by AZA leads to double-stranded RNA formation, recognized by innate anti-viral immune responses.

Next, we evaluated whether AZA-induced EREs also trigger innate immune responses in AML patients. Using the RNA-seq data of the Leucegene cohort (437 patients), we quantified the expression of the 506 ERE transcripts (Table S3) upregulated by AZA in our cell lines. Patients were segregated based on their cumulative expression of these EREs, and those expressing above-median levels (*n* = 219) were compared with those expressing below-median levels (*n* = 218). As expected, DEGs and GO analyses revealed that high levels of AZA-induced EREs were associated with upregulated defense responses against viruses (Fig. S6A). Furthermore, ERE expression levels significantly correlated with the expression of two critical double-stranded RNA response regulators, RIG-I and MDA5 (Fig. S6B). This shows that AZA-induced ERE expression is also associated with innate immune responses in vivo.

To complement our previous analysis, we also performed GO analyses on genes downregulated by AML patients expressing high levels of AZA-induced EREs. This showed that multiple pathways controlling proliferation were downregulated, suggesting that high ERE expression (and anti-double-stranded RNA response) might impact the growth of AML blasts (Fig. S6C). Unexpectedly, we also observed that many GO terms related to protein degradation/catabolism and autophagy were significantly downregulated in these patients. Since ERE RNAs can trigger autophagy [35] and be degraded by the autophagic process [35, 36], we hypothesized that enhanced autophagy in low-ERE expressing blasts could protect them from the deleterious effects that EREs have on their proliferation. Therefore, we next investigated whether AZA-treated cells present significant alterations in their protein homeostasis.

### AZA molds the immunopeptidome and induces protein aggregation through DNMT2 inhibition

**Fig. 4:**
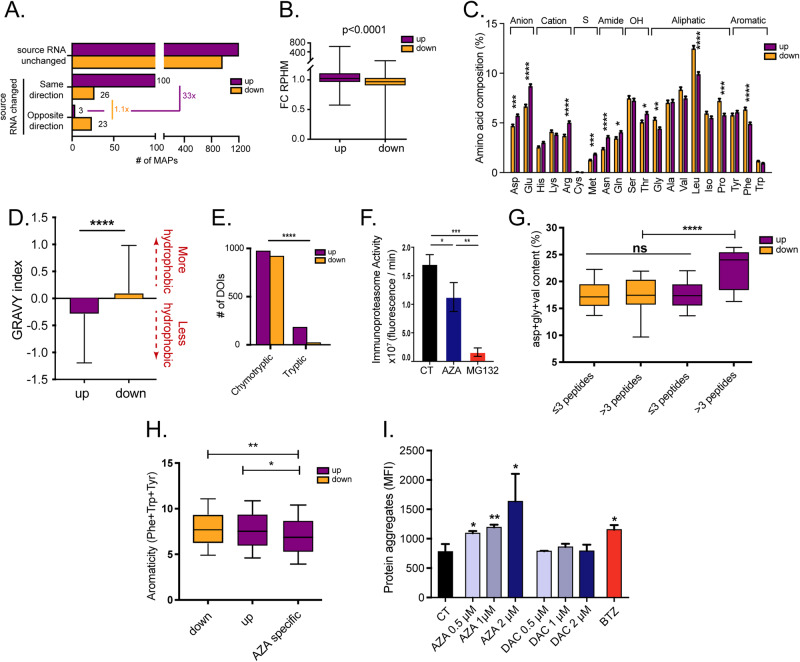
AZA molds the immunopeptidome through DNMT2 inhibition. THP-1, MOLM-13, SKM-1, and OCI-AML3 cells were treated for 72 h with 0.5 µM of AZA (supplemented at 0, 24, and 48 h), washed, and maintained in fresh medium for 24 h before analysis. **A** Bar plots depicting the number of up- and downregulated DEMs identified in all cell lines and variations in the expression of their source RNA. **B** Fold changes (FC) of reads per hundred million (rphm) expression of source transcripts generating up- and downregulated DEMs identified in all cell lines. **C** Amino-acid composition of up- and downregulated DEMs of interest identified in all cell lines. **D** Hydrophobicity of up- and downregulated DEMs of interest identified in all cell lines assessed by their GRAVY index. Scores > 0 reflect higher hydrophobicity. **E** Number of DEMs of interest (DOIs) identified in all cell lines associated with chymotryptic or tryptic activities based on their C-termini amino acid composition (Fisher’s exact test). **F** Immunoproteasome activity in OCI-AML3 cells after 0.5 µM AZA treatment for 24 h. MG132 (100 µM), a proteasome inhibitor, was used as a negative control (unpaired *t*-test). **G** Proportion of DNMT2-target amino acids (glycine (gly), valine (val), and aspartic acid (asp)) in proteins having generated MAPs among the up- or downregulated DEM fractions identified in all cell lines. **H** Aromaticity (frequency of phenylamine (Phe), tryptophan (Trp), and tyrosine (Tyr) residues) of proteins having generated AZA-specific (identified only in AZA condition) MAPs or up- and downregulated DEMs identified in all cell lines. **I**, Quantification of protein aggregates induced with increasing concentrations of AZA and DAC in OCI-AML3 cells after 24 h. Bortezomib (BTZ, 10 nM) was used as a positive control (unpaired *t*-test; ****p < 0.0001, ***p < 0.001, **p < 0.01, *p < 0.05).

The immunopeptidome is shaped by modifications in protein homeostasis [32] and by fluctuations in the abundance of transcripts [29, 37]. Therefore, we investigated whether immunopeptidomic changes in AZA-treated cells can be explained solely by variations in the transcriptome. Using BamQuery, a computational tool that quantifies the RNA expression of any MAP of interest [38], we observed that most AZA-altered DEMs displayed no change at the RNA level (Fig. 4A). Nevertheless, among DEMs coded by DEGs, RNA upregulation strongly correlated with concurrent upregulation of the corresponding DEMs (Fig. 4A). This was not the case for downregulated transcripts. Moreover, fold-changes in RNAs generating upregulated DEMs were significantly higher than those in downregulated DEMs (Fig. 4B). Altogether, these data show that RNA transcript upregulation does not fully explain DEM variations.

To gain insights into the protein homeostasis alterations affecting the immunopeptidome, we focused on DEMs whose source RNA fold-change did not explain their immunopeptidomic fold-change in the next sections. We started by analyzing their residue composition. Upregulated peptides contained more polar residues and presented a lower hydrophobicity than downregulated ones (Fig. 4C, D). Interestingly, hydrophobic residues are the preferential cleavage sites of proteasomes, particularly immunoproteasomes [39]. Furthermore, MAP generation by constitutive proteasomes depends mainly on their tryptic and chymotryptic-like activities, and chymotryptic-like activity is further amplified in immunoproteasomes [40, 41]. Hence, we assessed how protease activity contributed to the immunopeptidome by examining the C-terminal residue of each peptide. We observed that upregulated peptides resulted primarily from tryptic cleavage (Fig. 4E). Consistent with this, AZA treatment significantly reduced immunoproteasome activity (Fig. 4F, Fig. S7A).

Typically, alterations in proteasomal activity are associated with disrupted protein homeostasis [42, 43]. Therefore, we examined the residue composition of proteins that generated DEMs. Assuming that proteins generating multiple peptides are degraded more actively than those generating a single one, we correlated the number of DEMs for each protein with its amino acid composition. This analysis showed that aspartic acid (Asp) and glycine (Gly) had the best positive correlation with the number of upregulated DEMs (Table S4). Interestingly, the transfer RNAs of Asp and Gly are stabilized by DNMT2, a transfer RNA-methyl transferase enzyme inhibited by AZA [44, 45]. A comparison of the frequency of Asp, Gly, and valine (Val, the third amino acid whose transfer RNA is methylated by DNMT2) revealed that proteins generating more than three upregulated DEMs present significantly higher cumulative frequencies of Asp, Gly, and Val (Fig. 4G). Demethylated transfer RNAs are susceptible to ribonuclease cleavage and fragmentation [45]. Therefore, we hypothesized that AZA-mediated DNMT2 inhibition results in an insufficiency of Asp, Gly, and Val transfer RNAs and leads to a decrease in protein synthesis, ribosomal stalling, and consequent protein aggregate generation during translation of proteins rich in the aforementioned residues. Accordingly, AZA-specific peptides were generated from proteins with a significantly lower proportion of aromatic residues, a feature often associated with less efficient protein folding (Fig. 4H) [46]. Furthermore, we experimentally observed that AZA induces protein aggregates in a dose-dependent manner (Fig. 4I and Fig. S7C), and that DAC, which inhibits DNMT1 but not DNMT2, did not induce protein aggregates. Finally, siRNA knock-down of DNMT2 increased protein aggregation levels, in agreement with previous studies (Fig. S7B) [47]. Altogether, these results show that the immunopeptidome of AZA-treated cells is significantly impacted by the DNMT2 inhibition mediated by AZA.

### Autophagy degrades AZA-induced EREs

**Fig. 5:**
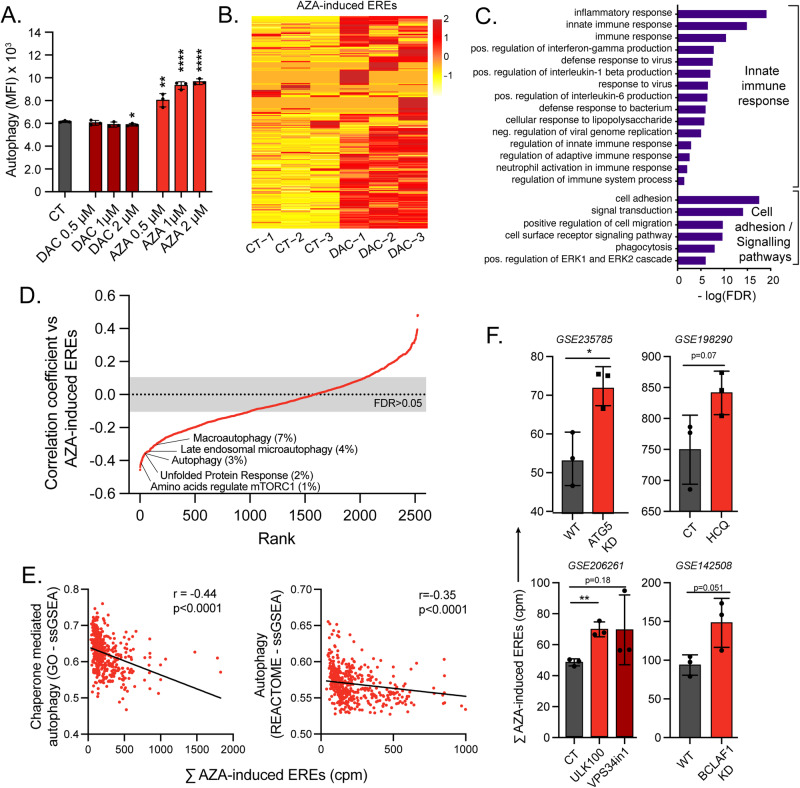
Autophagy degrades AZA-induced EREs. **A** THP-1 cells undergoing autophagy were assessed with increasing doses of AZA and DAC by flow cytometry for 24 h using specific autophagy detection fluorescent probes. **B** EREs commonly induced by AZA in our cell lines were quantified in RNA-seq data of DAC-treated THP-1 cells. **C** Histogram depicting GO-term analysis of the most significantly enriched biological processes associated with upregulated DEGs in THP-1 cells post-DAC treatment. **D** The expression of all REACTOME gene sets was determined in all patients of the Leucegene cohort by ssGSEA. Each of these gene sets was then correlated (Spearman) with the summed expression of the EREs induced by AZA in our cell lines. Gene sets were ranked in function of the correlation coefficients, and ranks were plotted vs the correlation coefficients. Some gene sets among the top 10% of ranks are highlighted. **E** Manual correlation between the indicated gene sets and the summed expression in counts per million (cpm) of the EREs found to be induced by AZA in our cell lines using single sample gene set enrichment analysis (ssGSEA). **F** Sum of the expression of the EREs found to be induced by AZA in our cell lines in indicated samples from the indicated RNA-seq experiments obtained from GEO (GSE identifications). KD=knock-down; HCQ=Hydroxychloroquine (autophagy inhibitor); ULK100 and VPS34in1 are autophagy inhibitors.

Given the inverse correlation between autophagy-related GO terms and EREs in AML patients and the generation of protein aggregates by AZA, we evaluated whether AZA induces autophagy. Twenty-four hours of treatment resulted in a dose-dependent induction of autophagy, assessed by an autophagy detection kit, in all AML cell lines (Fig. 5A and Fig. S8A). This agreed with previous reports [48–50] and was confirmed by western blot analysis of LC3-I/II (Fig. S8B). Interestingly, this autophagy induction was mainly absent with DAC, showing that autophagy induction depends on protein aggregates generation resulting from DNMT2 inhibition. As EREs were previously shown to trigger autophagy [35], we evaluated whether DAC induced the same EREs as AZA. Therefore, we treated THP-1 cells with a DAC dose (30 nM) that inhibited DNMT1 (Fig. S8C, D) without inducing cell death at 96 h, and observed that DAC upregulated the expression of the same EREs induced by AZA (Fig. 5B). This upregulation was concomitant with an induction of innate immune responses, evidenced by GO-term analysis (Fig. 5C and Table S5). This shows that the autophagy induced in AZA-treated (but not in DAC-treated) cells results from DNMT2 inhibition rather than ERE induction.

To further demonstrate that autophagy has a deleterious effect on ERE transcript levels, we performed a ssGSEA quantification of all REACTOME gene sets (*n* = 2528) in the Leucegene cohort (diagnosis samples). Next, we correlated each gene set with the expression of AZA-regulated EREs. A ranking of gene sets based on correlation coefficients evidenced that multiple gene sets related to autophagy and unfolded protein response were inversely correlated and among the top 10% of ranked gene sets, showing their importance in regulating ERE levels (Fig. 5D). Targeted correlations with gene sets from other sources and genes playing crucial roles in autophagy also showed similar results (Fig. 5E and S8E). This indicates that, at the time of diagnosis, AML cells with higher basal autophagic activity are associated with low ERE levels, and vice versa. Additionally, we downloaded data from previously published studies performing autophagy inhibitions [51, 52] and observed that autophagy inhibition, whether genetic or chemical, resulted systematically in the upregulation of EREs (Fig. 5F). Altogether, these results demonstrate that autophagy degrades ERE transcripts independently of AZA treatment.

**Fig. 6:**
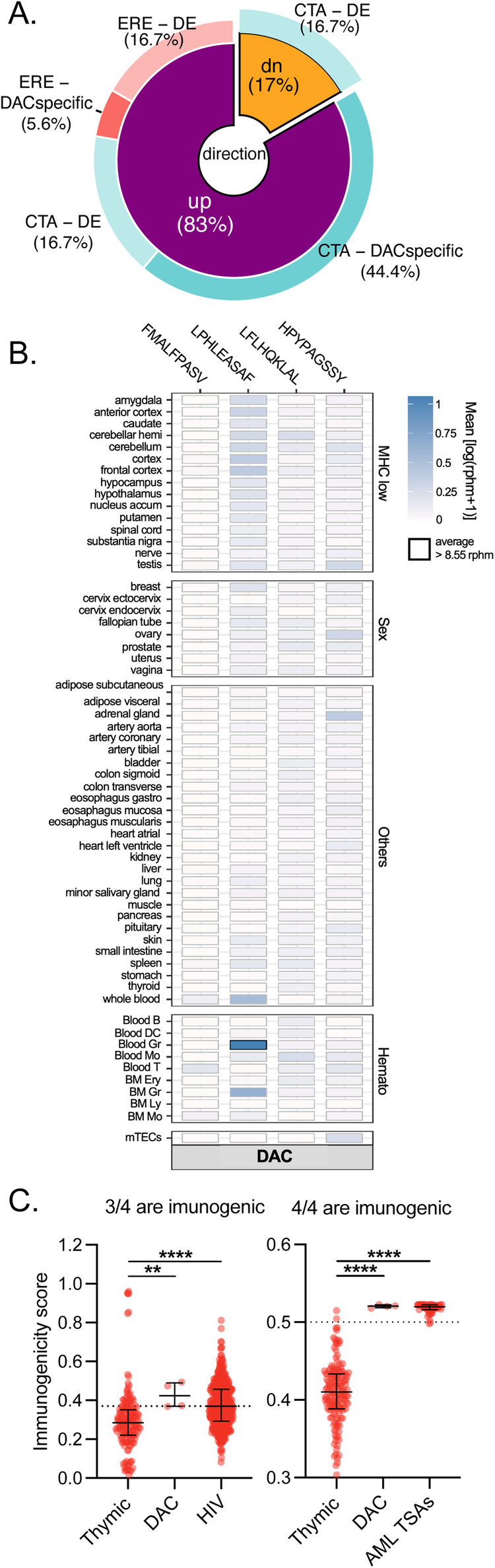
DAC induces immunogenic ERE MAPs. **A** Repartition of cancer-testis antigens (CTA) and ERE MAPs significantly altered by DAC in THP-1 cells. DE: differentially expressed, DACspecific: MAPs present in all three replicates of DAC and absent in CT. **B** Heatmap of average RNA expression of MAPs upregulated by DAC in normal tissues from GTEx (*n* = 12–50), normal hematopoietic cell populations (*n* = 3–16), or mTECs (*n* = 11). Boxes in which a MAP has an rphm > 8.55 are highlighted in black. **C** Comparison of immunogenicity scores (Repitope, left and BamQuery, right) among MAPs upregulated by DAC-treated THP-1 cells, MAPs from thymic stromal cells, AML tumor specific antigens (AML TSAs) and HIV MAPs. Thymic MAPs are negative control since they are not expected to be immunogenic, while AML TSAs and HIV MAPs are positive controls. Treatments refer to three days of daily 0.5 μM AZA or 30 nM DAC followed by 24-h discontinuation unless otherwise stated.

Finally, we tested whether autophagy induction would prevent the generation of MAPs by ERE transcripts. We, therefore, performed immunopeptidomic analyses on THP-1 cells treated with DAC. This revealed that in addition to cancer-testis MAPs, DAC upregulated the presentation of 4 ERE MAPs (Fig. 6A and Table S6), while none were downregulated. To estimate whether these MAPs could be recognized by CD8^+^ T cells, we queried their RNA expression in 50 normal human tissues from GTEx, in hematopoietic cells, and mTECs with BamQuery (Fig. 6B). Three out of four ERE MAPs were lowly or not expressed, suggesting the absence of central tolerance for these peptides [15, 38, 53]. Immunogenicity predictions with two machine learning algorithms (Repitope [54] and BamQuery [38]) further supported the immunogenicity of these MAPs (Fig. 6C). In summary, these data show that autophagy degrades ERE transcripts and hampers their MAP generation capacity.

### Autophagy inhibition has additive effects with AZA and increases AML immunogenicity

**Fig. 7:**
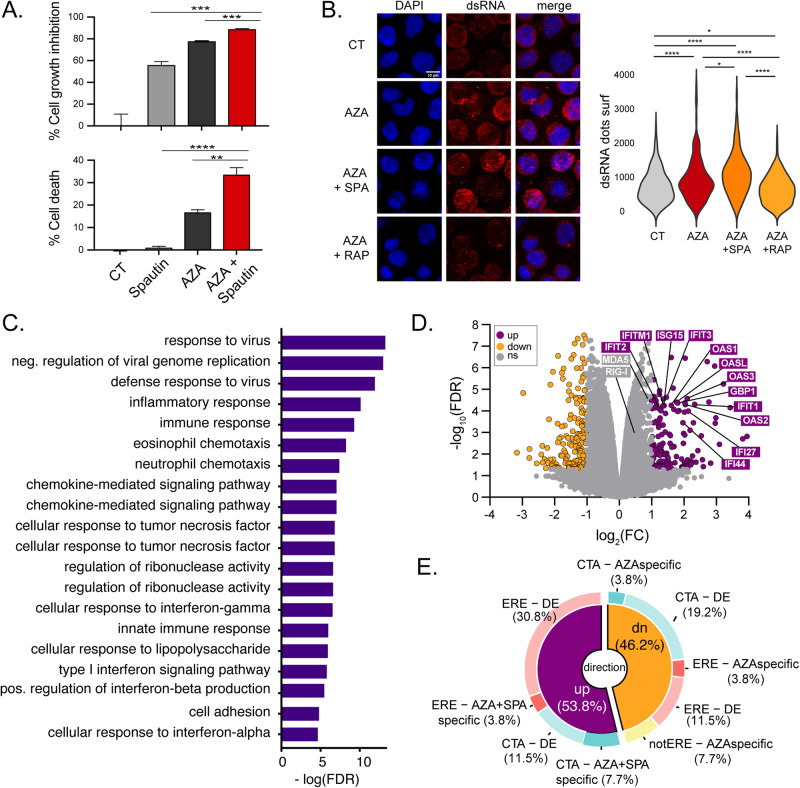
Autophagy inhibition synergizes with AZA by further increasing ERE expression. **A** Cell growth inhibition and cell death (7-AAD) of THP-1 cells treated either with AZA (1 μM), spautin-1 (5 μM), or both for four days. Control cells were treated with 0.1% DMSO in two independent experiments (unpaired *t*-test; ****p < 0.0001, ***p < 0.001, **p < 0.01). **B** Representative immunoflouresence images and quantification of double-stranded RNA (dsRNA) signals measured from microscopy images of THP-1 cells treated for four days with either Spautin-1, Rapamycin, or DMSO in the presence of 0.5 μM AZA (three days of daily AZA treatment followed by 24 h discontinuation). CT: 0.1% DMSO; AZA + SPA: AZA and 5 μM Spautin-1; AZA + RAP: AZA and 0.5 μM Rapamycin (unpaired *t*-test; *p < 0.05, ****p < 0.0001). **C** Histogram depicting GO-term analysis of the most significantly enriched biological processes associated with upregulated DEGs in THP-1 cells co-treated with AZA and Spautin-1. **D** Volcano plot of the differential gene expression analysis comparing THP-1 cells treated with AZA and AZA+Spautin-1 vs AZA only. Purple dots show genes upregulated (log2(fold-change, FC) > 1; FDR < 0.05) in AZA+Spautin-1 cells. MDA5 and RIG-I were significantly upregulated (FDR < 0.05) but below our cutoff threshold (log2(FC) > 1). **E** Distribution of cancer-testis antigens (CTA), ERE, and other non-canonical MAPs significantly altered by AZA and Spautin-1 vs. AZA alone. DE: differentially expressed, AZA+SPAspecific: MAPs present in all three replicates of the combination and absent in AZA only condition, AZAspecific: MAPs present in all three replicates of the AZA only condition and absent in combination.

Finally, we examined whether inhibiting autophagy would augment the anti-AML effect of AZA. THP-1 and OCI-AML3 cells were treated for three days with AZA and/or Spautin-1 (which inhibits autophagy and suppresses the unfolded protein response, the most likely process involved in autophagy induction in response to protein aggregates [53, 55]), and their survival and cell counts were evaluated. An additive effect between AZA and Spautin-1 was observed for both assays (Fig. 7A and Fig. S9A). While Spautin-1 alone reduced proliferation, it did not kill the cells (Fig. S9B, C), showing that autophagy is a survival mechanism upon AZA treatment. Similar results were obtained with another autophagy inhibitor, the VPS34 inhibitor SAR405 (Fig. S10).

Next, we investigated the effects of combined autophagy inhibition and AZA treatment on ERE-related responses. First, we either induced (with Rapamycin) or inhibited (with Spautin-1) autophagy in combination with AZA and measured double-stranded RNAs with microscopy. As expected, Rapamycin decreased while Spautin-1 increased their levels (Fig. 7B). Next, we sought to perform immunopeptidomic analyses to evaluate the effects of AZA+Spautin-1 co-treatment. Therefore, we determined a co-treatment regimen through which autophagy and DNMT1 are inhibited and the viability of the cells is maintained (See Methods and Fig. S11A, B). RNA-seq analyses confirmed that combining AZA and Spautin-1 induced greater levels of EREs compared to AZA alone (Fig. S11C). This increase was accompanied by an induction of anti-viral innate immune responses, notably illustrated by a significant upregulation of OAS and IFN-induced genes and GO-terms analysis (Fig. 7C, D and Table S7). This shows that autophagy inhibition can increase the expression of ERE transcripts, triggering innate immune responses.

**Fig. 8:**
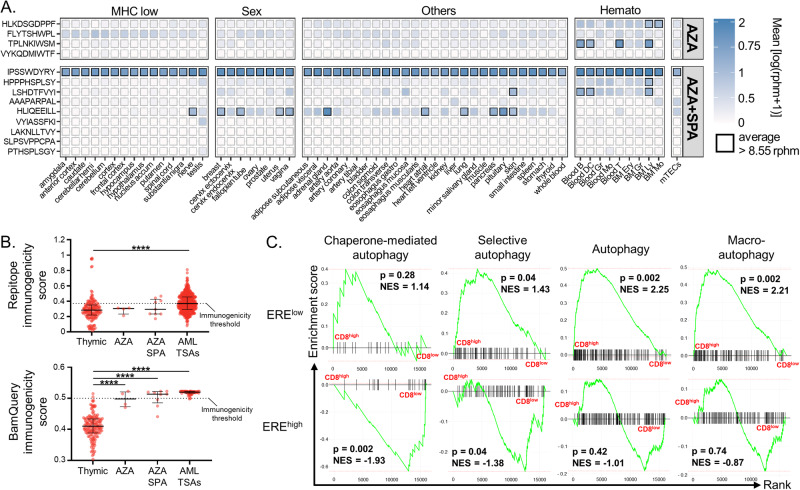
Autophagy inhibition promotes AZA’s generation of immunogenic ERE MAPs. **A** Heatmap of average RNA expression of MAPs downregulated (four on top) or upregulated (nine on bottom) after AZA+Spautin-1 vs AZA only treatment in normal tissues from GTEx (*n* = 12–50), normal hematopoietic cell populations (*n* = 3–16), or mTECs (*n* = 11). Boxes in which a MAP has an rphm >8.55 are highlighted in black. **B** Comparison of immunogenicity scores (Repitope and BamQuery) among MAPs downregulated (four on top in panel A) or upregulated (nine on bottom in panel A) after AZA+Spautin-1 vs AZA-only treatment, MAPs from thymic stromal cells, AML tumor specific antigens (AML TSAs) and HIV MAPs. Thymic MAPs are negative control since they are not expected to be immunogenic, while AML TSAs and HIV MAPs are positive controls. **C** GSEA of the indicated REACTOME gene sets in the indicated comparisons. ERE^high^ and ERE^low^: AML specimens expressing high and low levels of ERE respectively, NES: normalized enrichment score. AZA and Spautin-1 co-treatment experiments refer to treatment with Spautin-1 (5 μM) or DMSO (0.1%) for 24 h followed by 0.5 μM AZA for three days followed by 48 h discontinuation.

Next, we investigated whether autophagy inhibition could restore the generation of ERE MAPs after AZA treatment. Immunopeptidomic analyses of THP-1 cells treated with AZA alone or combined with Spautin-1 showed that the combination upregulated nine ERE MAPs, while four were downregulated (Fig. 7E). With 828 upregulated MAPs and 904 downregulated ones, this represented a ~ 2.5-fold increase in the proportion of ERE MAPs among the immunopeptidome (1.08% vs 0.44%, Table S8). This upregulation was noteworthy because our original experiments did not detect a single upregulated ERE-derived MAPs in AZA-treated THP-1 cells. Notably, five out of nine ERE MAPs induced by the combination were lowly expressed in normal tissues (vs two out of four downregulated ones) (Fig. 8A). Immunogenicity prediction tools showed that upregulated ERE MAPs are more immunogenic than downregulated ones (Fig. 8B). Indeed, four out of nine (with Repitope) and six out of nine (with BamQuery) ERE MAPs upregulated by AZA+Spautin-1-treated cells were above immunogenicity thresholds, while none (Repitope) and two out of four (BamQuery) downregulated ones were above thresholds. These data show that autophagy inhibition promotes AZA’s generation of immunogenic ERE MAPs.

Since autophagy inhibition increases immunogenic ERE MAPs, we reasoned that leukemic blasts with elevated autophagy might be less recognized by CD8^+^ T cells. To assess this, we segregated the Leucegene AML patients based on two parameters. The first was the expression of CD8A and CD8B transcripts, reliable markers of CD8^+^ T-cell abundance in RNA-seq [56]. The second was the count of highly expressed AZA-induced EREs (HE-EREs), i.e., the number of EREs whose expression is above their median RNA expression across all patients having a non-null expression of the given ERE (a metric aimed at reflecting the density and diversity of epitopes presented by leukemic blasts [29]) (Fig. S11D). A GSEA comparing CD8^high^ vs. CD8^low^ patients within ERE^high^ and ERE^low^ groups revealed that the presence of CD8^+^ T cells was associated with the same processes in ERE^high^ and ERE^low^ patients, except for two gene sets related to DNA repair/proliferation, three related to metabolism, and two related to unfolded protein response and mTORC1 signaling (Fig. S11E). As mTORC1 regulates autophagy, we performed additional GSEAs with four gene sets related to autophagy from the REACTOME database. We found that all four were inversely associated with the presence of CD8^+^ T cells in ERE^high^ patients (two significantly), while the opposite was found for ERE^low^ patients (Fig. 8C). Altogether, these results further support that autophagy prevents the generation of ERE-derived MAPs, thereby precluding AML blasts from being recognized by CD8^+^ T cells.

## Discussion

Due to their capacity to trigger innate immune responses and generate immunogenic MAPs [30], EREs represent attractive targets for developing new immunotherapeutic avenues [26, 29, 57, 58]. Although AZA has been proposed to promote anti-tumor CD8^+^ T-cell responses through the induction of cancer-testis antigens, the contribution of ERE MAPs to such responses remains elusive. Aiming to unravel this contribution, we performed a thorough proteogenomic investigation to uncover changes in the MAP repertoire after AZA treatment. As expected, we identified cancer-testis antigens upregulated at the transcriptomic and immunopeptidomic levels. In contrast, ERE MAP abundance remained unchanged after AZA treatment in the four cell lines examined. This suggests that T-cell-mediated responses post-AZA treatment are more likely due to the recognition of cancer-testis MAPs than EREs. A recent report analyzing the CD8^+^ T-cell subsets targeting ERE-derived MAPs revealed no increase in ERE-reactive T cells post-AZA treatment in myeloid hematological malignancies, further supporting our observations [59].

The virtual absence of ERE MAP induction by AZA was paradoxical. Indeed, AZA strongly induced ERE transcripts, and the processing of numerous EREs should generate MAPs [30]. In AML patients, the basal ERE expression was positively associated with the expression of molecules involved in double-stranded RNA detection and anti-viral immune responses. In a previous report, high ERE expression in primary AML cells was associated with a favorable prognosis [60]. In addition, the clinical responses to AZA in myelodysplastic syndrome and AML is associated with the expression of a specific class of EREs inducing innate immune responses [61]. Therefore, elevated ERE expression certainly benefits patients’ outcomes by inducing anti-double-stranded RNA immune responses, and maximizing these responses should be pursued.

Notably, we observed that ERE levels (and associated innate immune responses) were inversely correlated to the expression of autophagy molecules in AML patients, and enhanced autophagy was found in our AZA-treated cells. Upon examination of the immunopeptidomic changes, we could attribute this latter observation to AZA’s inhibition of DNMT2 activity. Indeed, previous studies have shown that transfer RNA methylation by DNMT2 is involved in Asp-transfer RNA codon fidelity, and its loss leads to the production of misfolded proteins [62]. Autophagy is increasingly implicated in resistance to anti-cancer therapies, including resistance to AZA [48]. Here, abrogating autophagy by combining AZA with Spautin-1 or using DAC (which did not induce autophagy) showed an increase in both ERE transcripts and MAPs. Accordingly, an article also exploring the immunopeptidomic effects of DAC in glioblastoma cell lines evidenced an induction of ERE MAPs [63]. Thus, we conclude that the autophagic process triggered by DNMT2 inhibition hampers the proper induction of the presentation of ERE MAPs after AZA treatment.

While little is known about the interplay between EREs (remnants of ancient viruses) and autophagy, it is well-reported that autophagy is a defense response against viruses. Following infection, autophagy is triggered by the signaling of pattern-recognition receptors (such as RIG-like receptors) [64] to sustain the presentation of intracellular source proteins by MHC-II molecules [65]. Thereby, autophagy inhibition reduces the presentation of viral peptides to CD4^+^ T cells [66, 67]. In contrast, autophagy inhibition tends to increase MHC-I expression and the capacity to induce antiviral CD8^+^ T cell responses [68, 69]. Furthermore, autophagy can limit RIG-I-dependent IFN production by disrupting its signaling cascade [70, 71]. While these studies point to a potential negative correlation between autophagy and MHC-I presentation, they do not provide a precise molecular mechanism explaining how AZA-induced autophagy could prevent the generation of ERE MAPs. While this question will need to be explored in future studies, we surmise that autophagy degrades ERE RNAs instead of degrading their translational product. Indeed, autophagy has been reported to target viral [72] and ERE [35, 36] double-stranded RNAs to autophagosomes. Notably, this would explain why cancer-testis MAPs (which do not result from double-stranded RNAs) were successfully presented at higher levels after AZA treatment.

In conclusion, our results demonstrate that AZA-induced autophagy mitigates the ERE-dependent immune effects of AZA. Moreover, our results show that autophagy inhibition could be a desirable therapeutic option to combine with AZA. Adding autophagy inhibitors to AZA would have three desirable consequences: (1) to increase the direct cytotoxicity of AZA by preventing AML adaptation to proteotoxic stress (in agreement with results from [50]), (2) to increase ERE transcripts abundance and the subsequent beneficial innate immune responses and (3) to improve ERE MAP presentation and thereby adaptive T-cell responses. Furthermore, some of these ERE MAPs are predicted to be immunogenic, making them attractive tumor vaccine candidates, and further investigations should be pursued in this direction.

### Supplementary information

Supplementary Figures and Methods

Supplemental Table 1

Supplemental Table 2

Supplemental Table 3

Supplemental Table 4

Supplemental Table 5

Supplemental Table 6

Supplemental Table 7

Supplemental Table 8

## Data Availability

The accession number for the RNA sequencing and expression data reported in this paper is GEO:GSE217572 and GSE248580. MS raw data and associated databases are deposited to the ProteomeXchange Consortium via the PRIDE partner repository with the following dataset identifiers: PXD038663 and PXD046853. The code necessary for the generation of MS databases has been deposited on Zenodo 10.5281/zenodo.7096388. The remaining datasets generated during and/or analysed during the current study are available from the corresponding author on reasonable request.
