# Supplementary material for: Autophagy degrades immunogenic endogenous retroelements induced by 5-azacytidine in acute myeloid leukemia
Source: Leukemia. 2024 Apr 16;38(5):1019–31. doi: 10.1038/s41375-024-02250-6 (PMC11073987; doi:10.1038/s41375-024-02250-6)
Supplement: Supplementary file 1 — Supplementary Figures and Methods [file 41375_2024_2250_MOESM1_ESM.pdf]

Autophagy degrades immunogenic endogenous retroelements induced by 5-azacytidine in acute myeloid leukemia

Nandita Noronha[1], Chantal Durette[1], Maxime Cahuzac[1], Bianca E Silva[2], Justine Courtois[2], Juliette Humeau[1], Allan Sauvat[3], Marie-Pierre Hardy[1], Krystel Vincent[1], Jean-Philippe Laverdure[1], Joël Lanoix[1], Frédéric Baron[2], Pierre Thibault[1,4], Claude Perreault[1,4], Gregory Ehx[1,2,4,5].

[1] IRIC, University of Montreal, Montreal, Canada. [2] GIGA-I3: Hematology, University of Liege, Liege, Belgium, [3] Equipe labellisée par la Ligue contre le Cancer, Université de Paris, Sorbonne Université, Inserm U1138, Institut Universitaire de France, Paris, France. [4] Senior authors. [5] Lead contact

***Correspondence**: g.ehx@uliege.be (G.E.)

Supplementary Figures and Methods

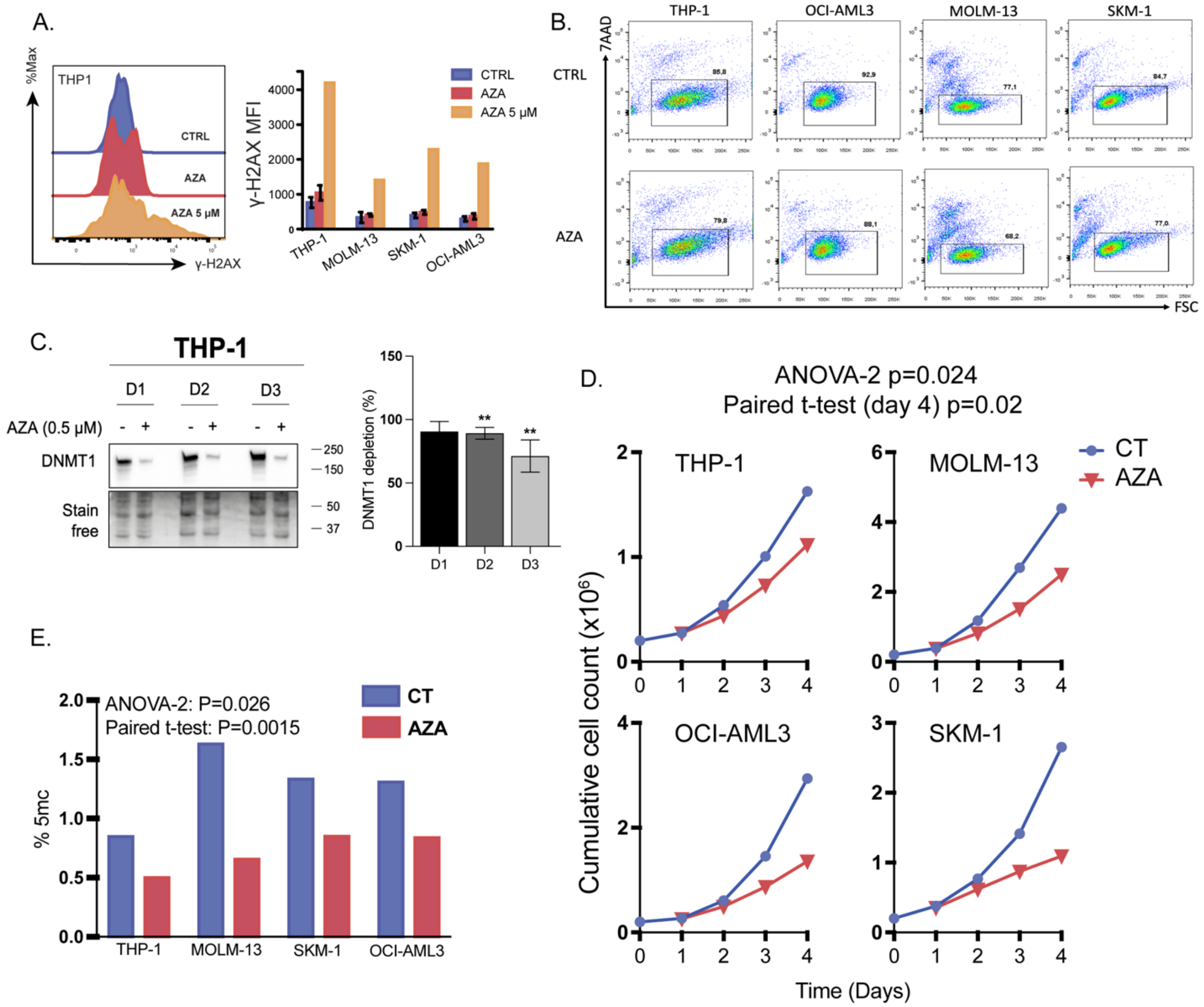

Figure S1. Low AZA treatment reduces DNA methylation and cell growth without inducing major cytotoxic effects from DNA damage in AML cell lines

A, Low AZA doses were added to four AML cell lines daily for three days (0.25 µM: MOLM-13 and SKM-1; 0.5 µM: THP-1 and OCI-AML-3), and the formation of DNA double-strand breaks was monitored by flow cytometry by measuring histone H2AX phosphorylation. The left panels depict representative histograms of THP-1 cells, while the right panels depict bar plots summarizing the percentage of expression of all four AML cells. Percentages were calculated by comparing AZA-treated cells to control cells. A high AZA dose (5 µM) was used as a positive control for double-strand break formation. **B,** Flow cytometry analysis (representative plots) of the viability (7-AAD negative cells) after AZA treatment in all four tested cell lines. **C,** Western blot analysis using stain-free gels of DNMT1 levels in THP-1 cells. (representative blots of two independent analyses, unpaired t-test, ** p<0.01) **D**, Cell growth of four AML cell lines was monitored after AZA treatment by counting 7-AAD negative cells via flow cytometry

E, 5-methylcytosine levels measured by ELISA with the MethylFlash Global DNA Methylation Kit after AZA treatment in AML cell lines.

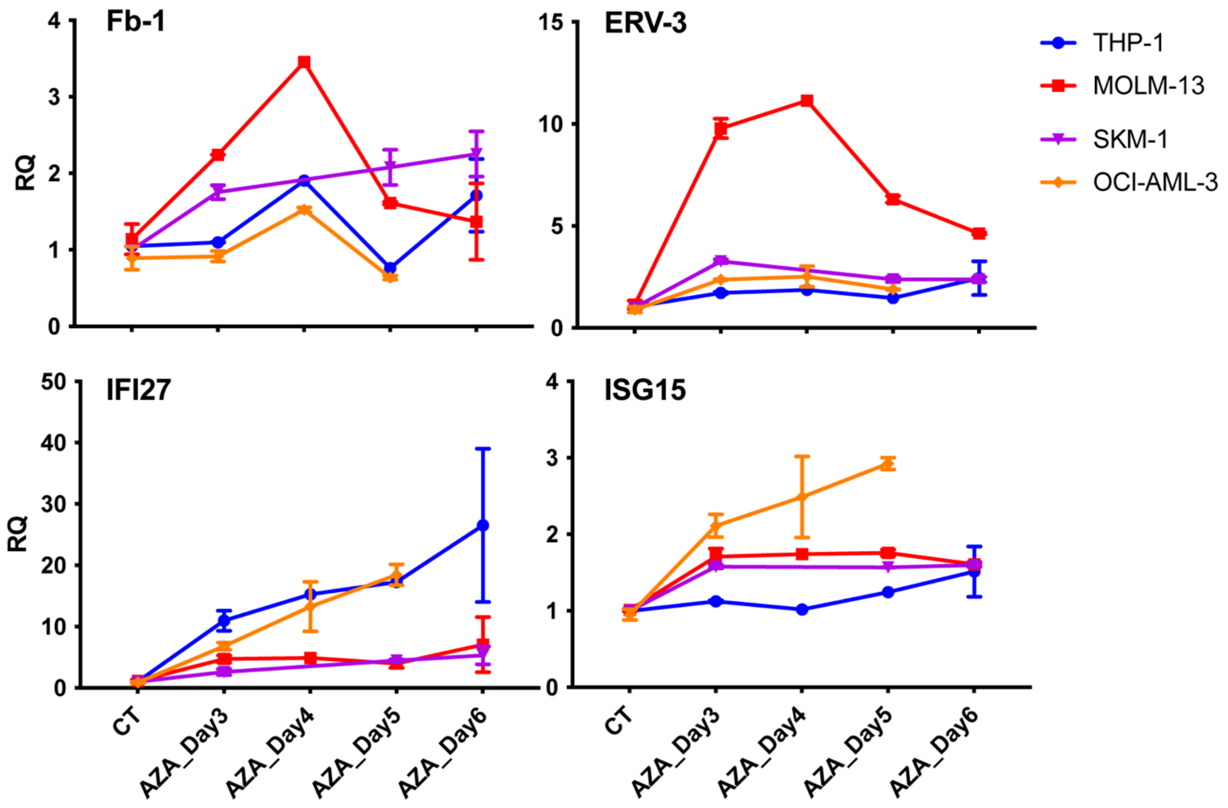

Figure S2. Low AZA treatment leads to delayed, transient ERE and dsRNA-induced pro-inflammatory gene expression in AML cell lines

Relative quantification levels of ERE (upper panel) and dsRNA-induced interferon (lower panel) gene candidates in four AML cell lines monitored by qPCR after AZA treatment for three days, followed by AZA discontinuation.

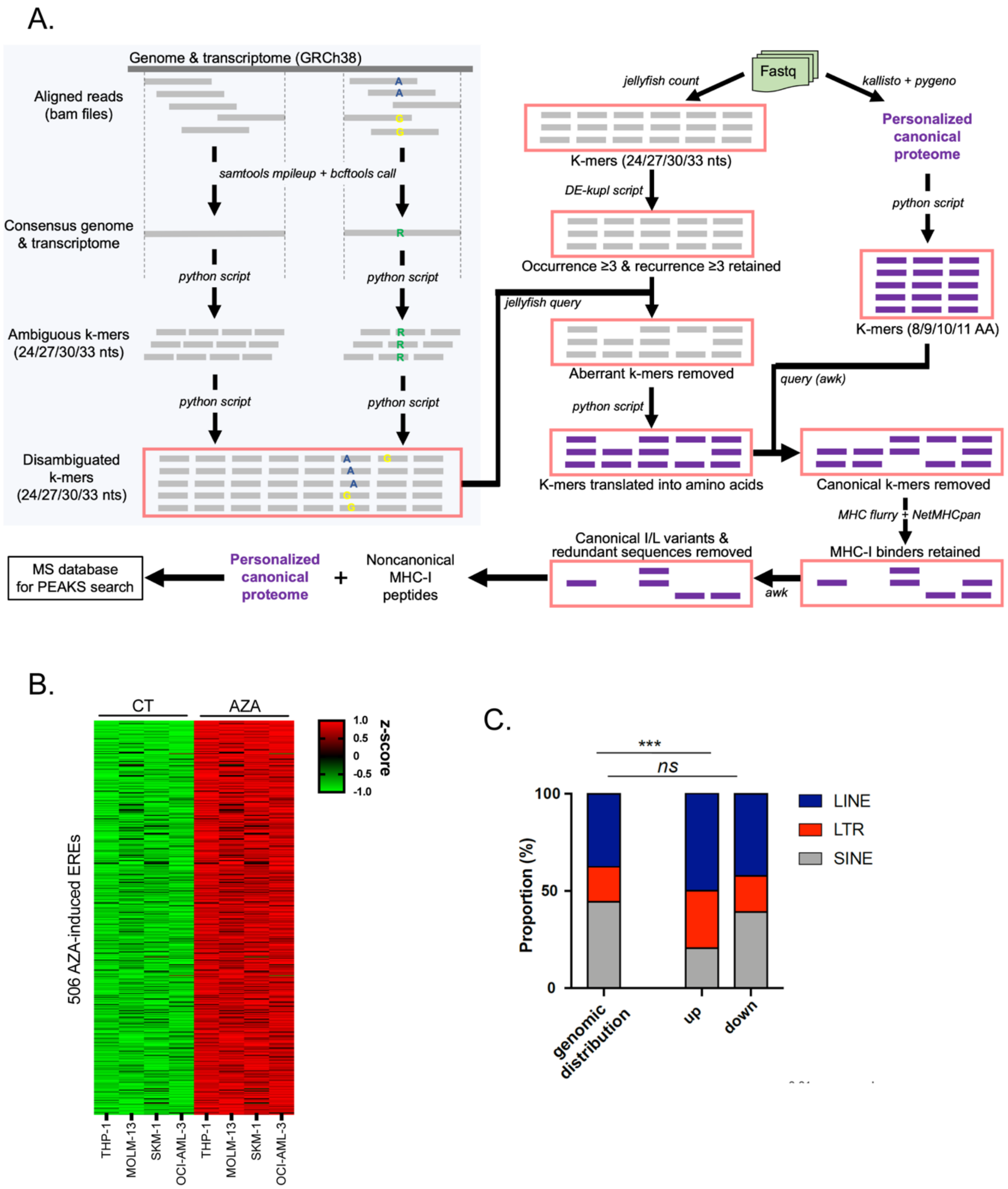

Figure S3. AZA-induced EREs do not generate MAPs but trigger innate immune responses **A,** Detailed proteo-genomic pipeline used for database generation for MS analyses **B,** Heatmap showing the expression (z-score normalization) of all 506 EREs significantly upregulated by AZA across the four tested cell lines (three days daily 0.5 µM AZA treatment followed by 24 hours discontinuation). **C**, Stacked bar plots of ERE group distribution at the genomic and transcriptomic levels for commonly up- and downregulated AZA-altered ERE sequences in all four cell lines.

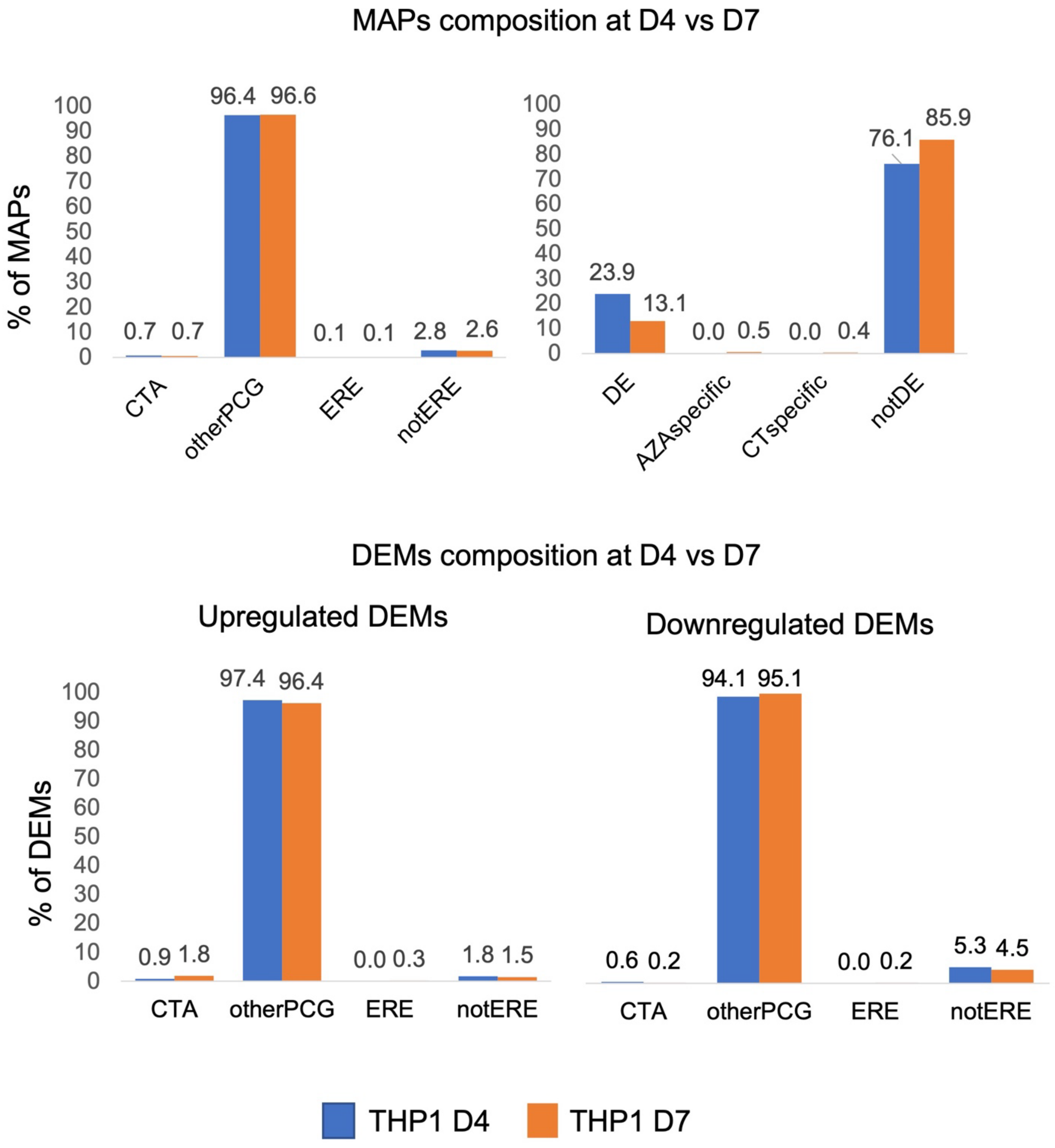

Figure S4. Immunopeptidomic analyses at a later time-point reveal no increase in ERE-derived DEMs

Comparison of MAP (upper panels) and DEM (lower panels) composition on days 4 and 7 in THP-1 cells. THP-1 cells were treated with 0.5 µM daily for three days followed by discontinuation for 24 hours or 96 hours (thus, analysis on day 4 or day 7, respectively). OtherPCG: other protein coding genes, DE: differentially expressed.

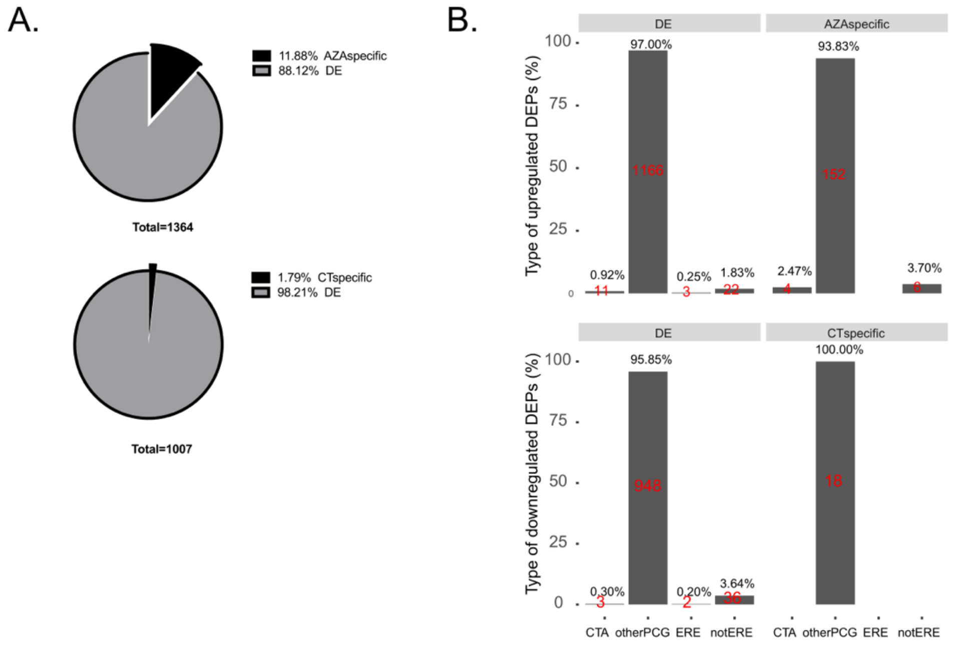

Figure S5. MAPs presented *de novo* after treatment derived from CTAs rather than EREs
A, Pie charts indicating the proportion of new MAPs previously unidentified on untreated cells (AZA specific) or MAPs unidentified after AZA treatment (CT specific) and differentially expressed for up- (upper panel) and downregulated DEMs (lower panel). **B**, Bar plots indicating DEM composition according to biotypes for up- (upper panel) and downregulated DEMs (lower panel). AZA treatment refers to 0.5 μM daily for three days followed by discontinuation for 96 hours in THP-1 cells. OtherPCG: other protein coding genes, DE: differentially expressed.

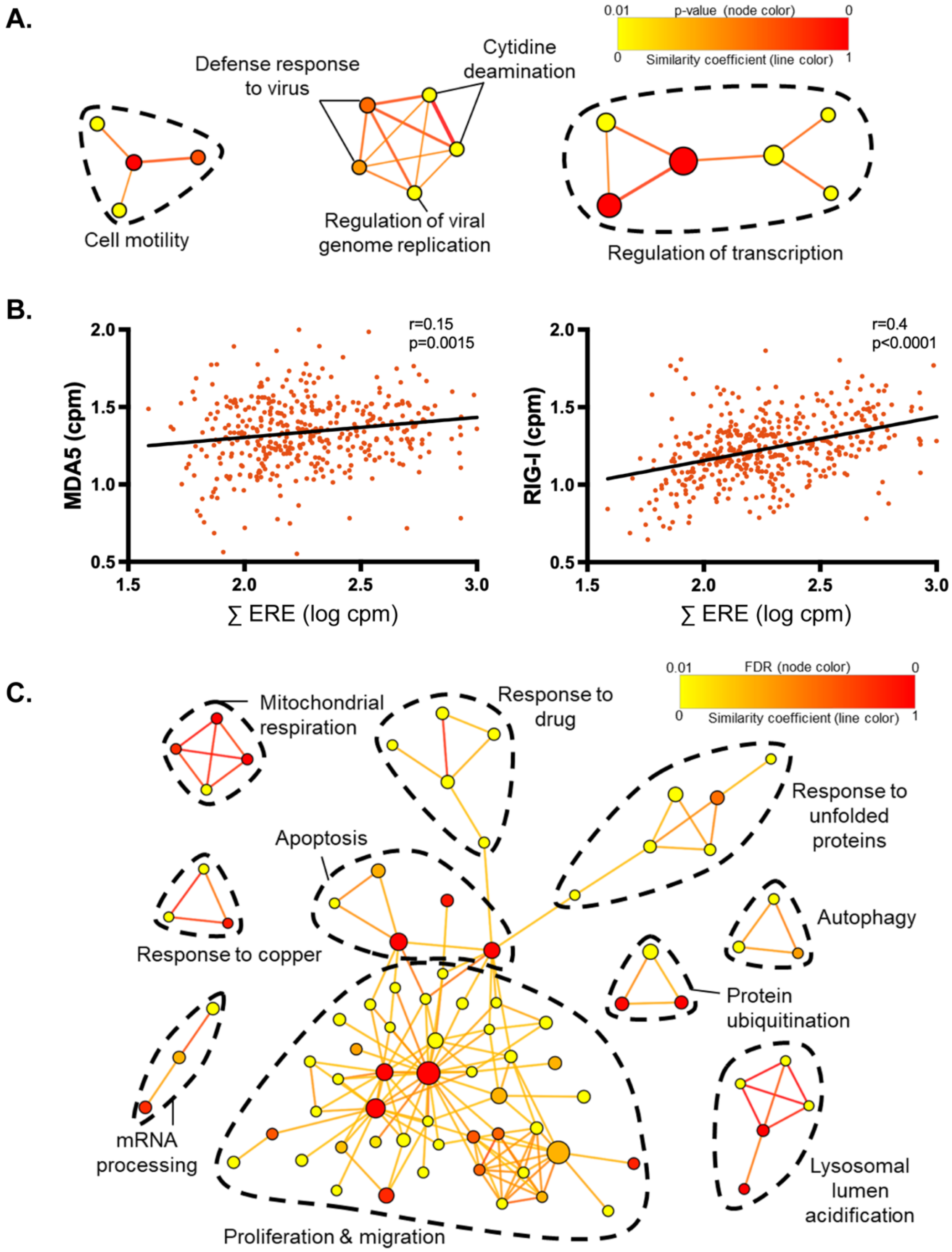

Figure S6. AZA-induced EREs trigger innate immune responses in primary AML specimens
A, Network analysis of GO-terms in AML patients (Leucegene cohort; n=437) expressing high levels of EREs induced by AZA in our four cell lines. **B**, Pearson correlation between genes involved in anti-dsRNA responses (MDA-5 and RIG-I) and AZA-induced EREs in AML patients. **C**, Network analysis of GO-terms enriched in patients expressing low levels of AZA-induced EREs. In B, and D, the line color reflects the similarity coefficient between connected nodes. Node color reflects the false discovery rate (FDR) of the enrichment. Node size is proportional to gene set size.

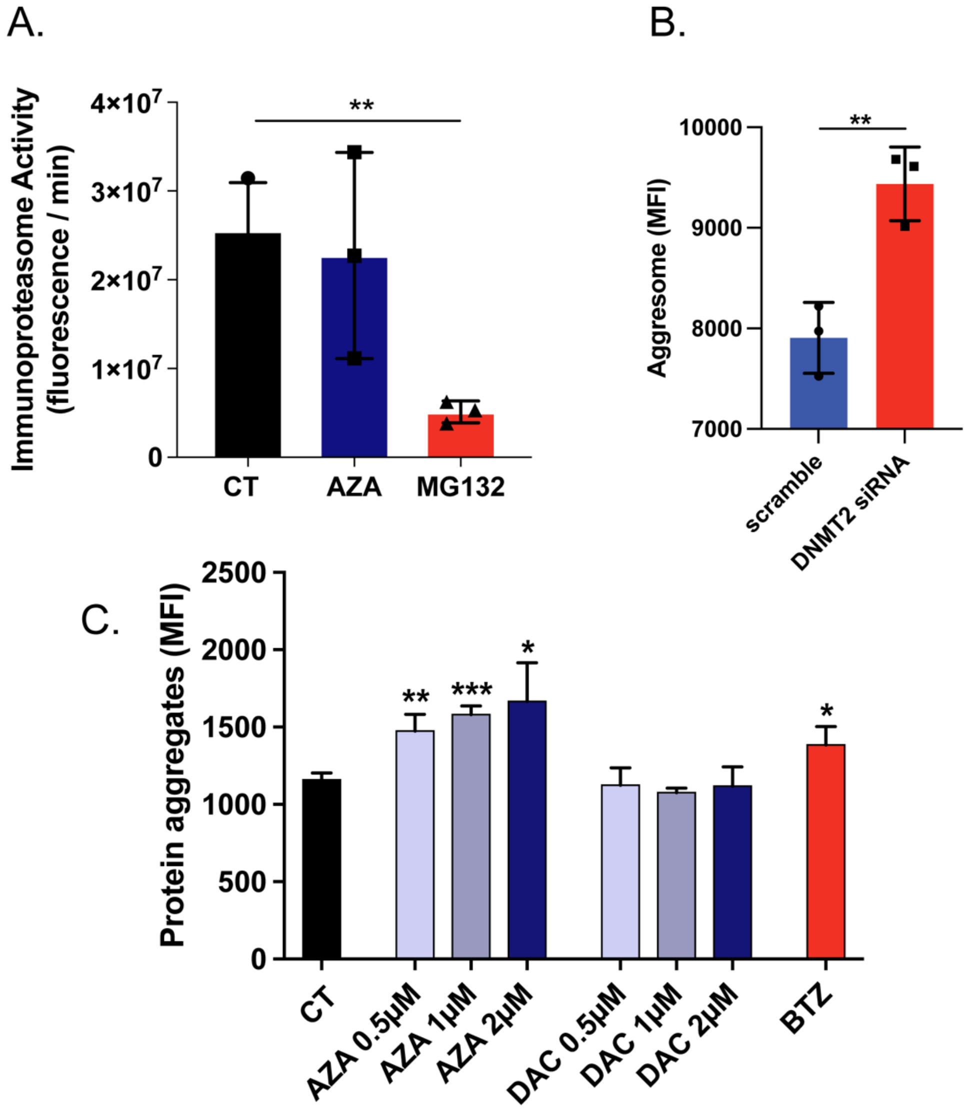

Figure S7. AZA molds the immunopeptidome through DNMT2 inhibition

A, Immunoproteasome activity monitored in THP-1 cells after 0.5 µM AZA treatment. MG132 (100 µM), a proteasome inhibitor, was used as a negative control (unpaired t-test; ** p< 0. 01) **B**, THP-1 cells were transfected with DNMT2-targeting siRNA or with a control siRNA. Protein aggregates were measured with the Proteostat kit, 48h after the transfection. (unpaired t-test; **p<0.01) **C**, Quantification of protein aggregates induced with increasing AZA and DAC concentrations in THP-1 cells treated for 24 hours. Bortezomib (BTZ, 100 µM) was used as a positive control (unpaired t-test; *** p< 0.001, * p< 0.05).

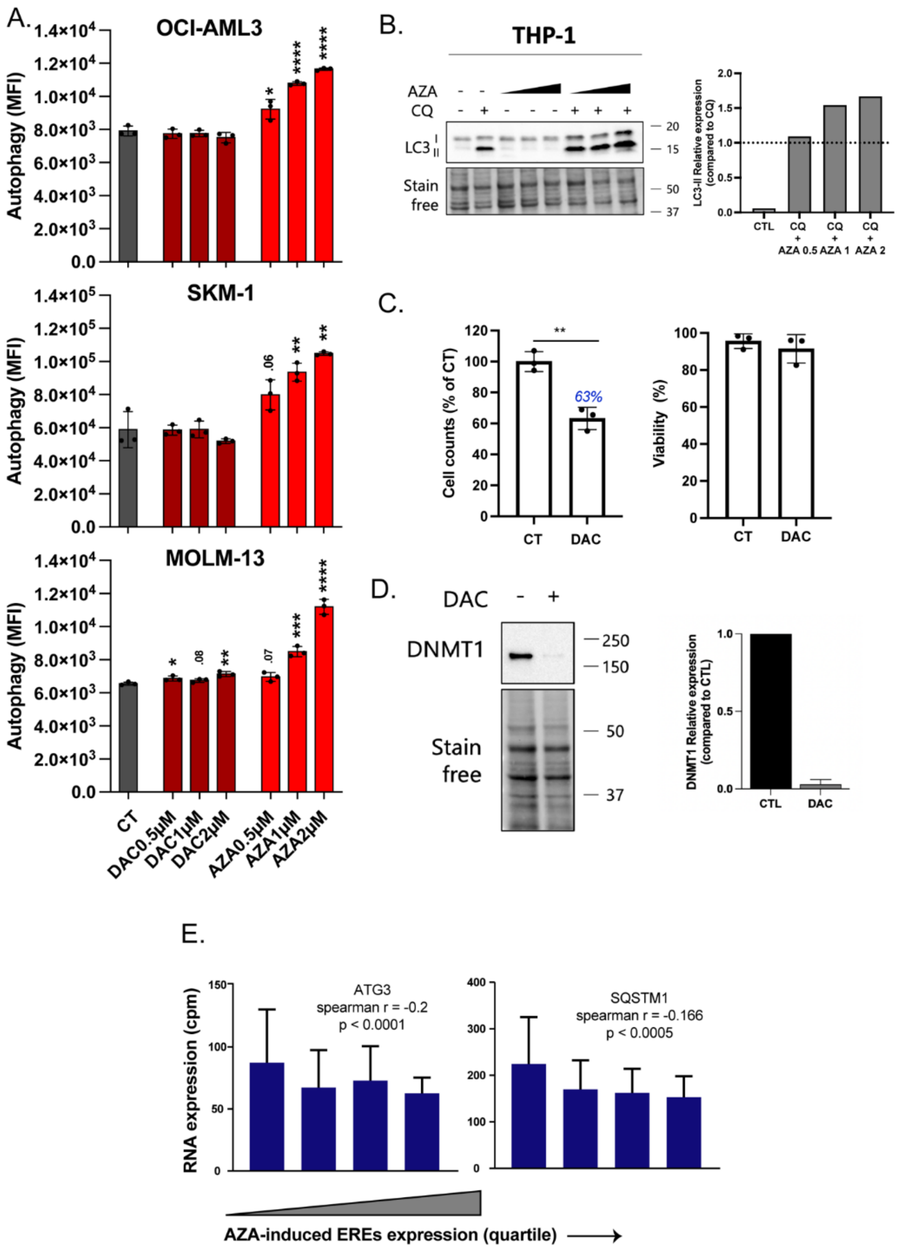

Figure S8. Autophagy degrades AZA-induced EREs

A, OCI-AML3, SKM-1, and MOLM-13 cells undergoing autophagy were assessed with increasing doses of AZA and DAC for 24 hours by flow cytometry using specific autophagy detection fluorescent probes (unpaired t-test; **** p< 0.0001,*** p< 0.001,** p< 0.01, * p< 0.05). **B**, Western blot analysis of LC3-II protein levels in THP-1 cells after treatment with increasing doses of AZA for 24 hours in the presence or absence of chloroquine (CQ) (representative of two independent experiments) **C**, Cell counts and viability of THP-1 cells treated with 30 nM DAC or DMSO, monitored with 7-AAD via flow cytometry (three independent experiments; unpaired t-test; ** p<0.01) **D,** Western blot analysis of DNMT1 protein levels in THP-1 cells after treatment with 30 nM DAC or DMSO (three independent experiments; unpaired t-test; *** p< 0.001) **E,** Bar plots of mean RNA expression (in cpm) of key autophagy genes (ATG3 and SQSTM1) in Leucegene AML patients segregated into quartiles based on AZA-induced ERE expression. Spearman correlations were computed without this segregation.

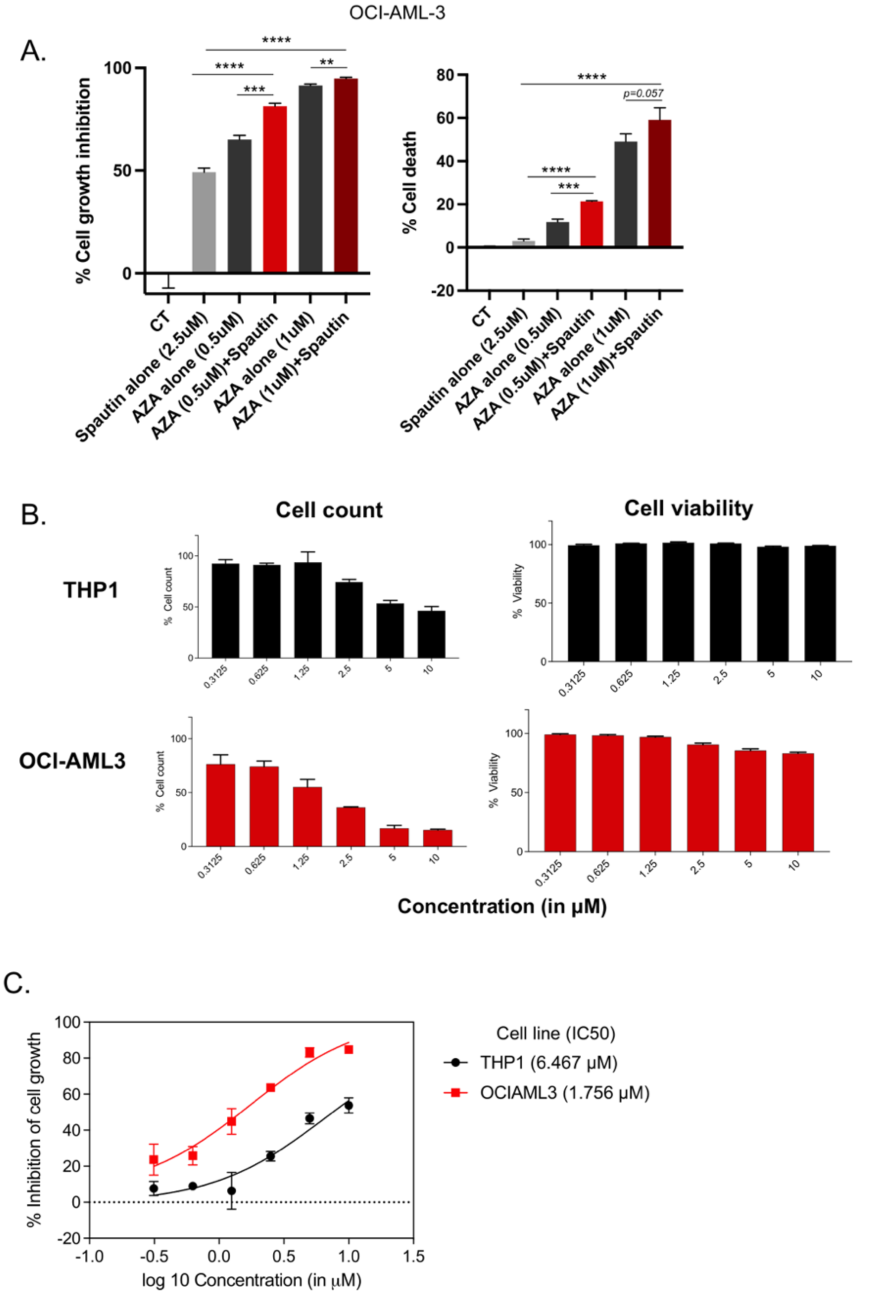

Figure S9. Autophagy inhibition synergizes with AZA, and spautin-1 treatment alone does not induce cell death

A, Cell growth inhibition and cell death of OCI-AML-3 cells treated with either increasing concentrations of AZA, Spautin-1, or both, monitored with 7-AAD via flow cytometry. Control cells were OCI-AML-3 cells treated with 0.1% DMSO (two independent experiments; unpaired t-test; **** $p < 0.0001$, *** $p < 0.001$, ** $p < 0.01$). **B**, Viable cell counts after treatment with increasing concentrations of Spautin-1 in THP-1 and OCI-AML-3 compared to DMSO-treated control cells using 7-AAD via flow cytometry. **C**, Dose-response curves and IC50 values generated from B.

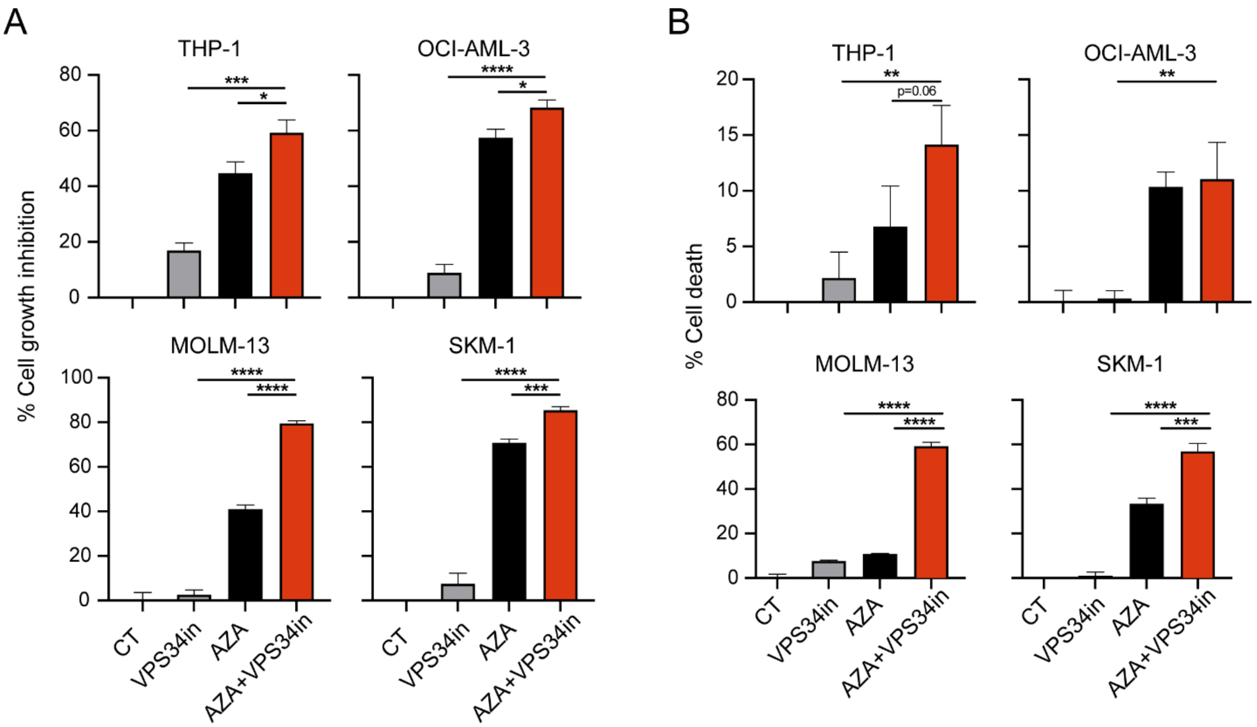

Figure S10. Autophagy inhibition with VPS34 inhibitor synergizes with AZA.
Indicated cell lines were cultured with AZA (1µM, added at 0, 24 and 48h) and the VPS34 inhibitor SAR405 (1µM, added at 0 and 48h). **A,** Cell growth (100-(#viable cells in considered well / average of viable cells in the three untreated wells*100)) was monitored by flow cytometry at 96h (7-AAD staining). **(B)** Viability (%7-AAD negative cells among the total cell population) was evaluated at 96h. Unpaired t-tests were used for all comparisons.

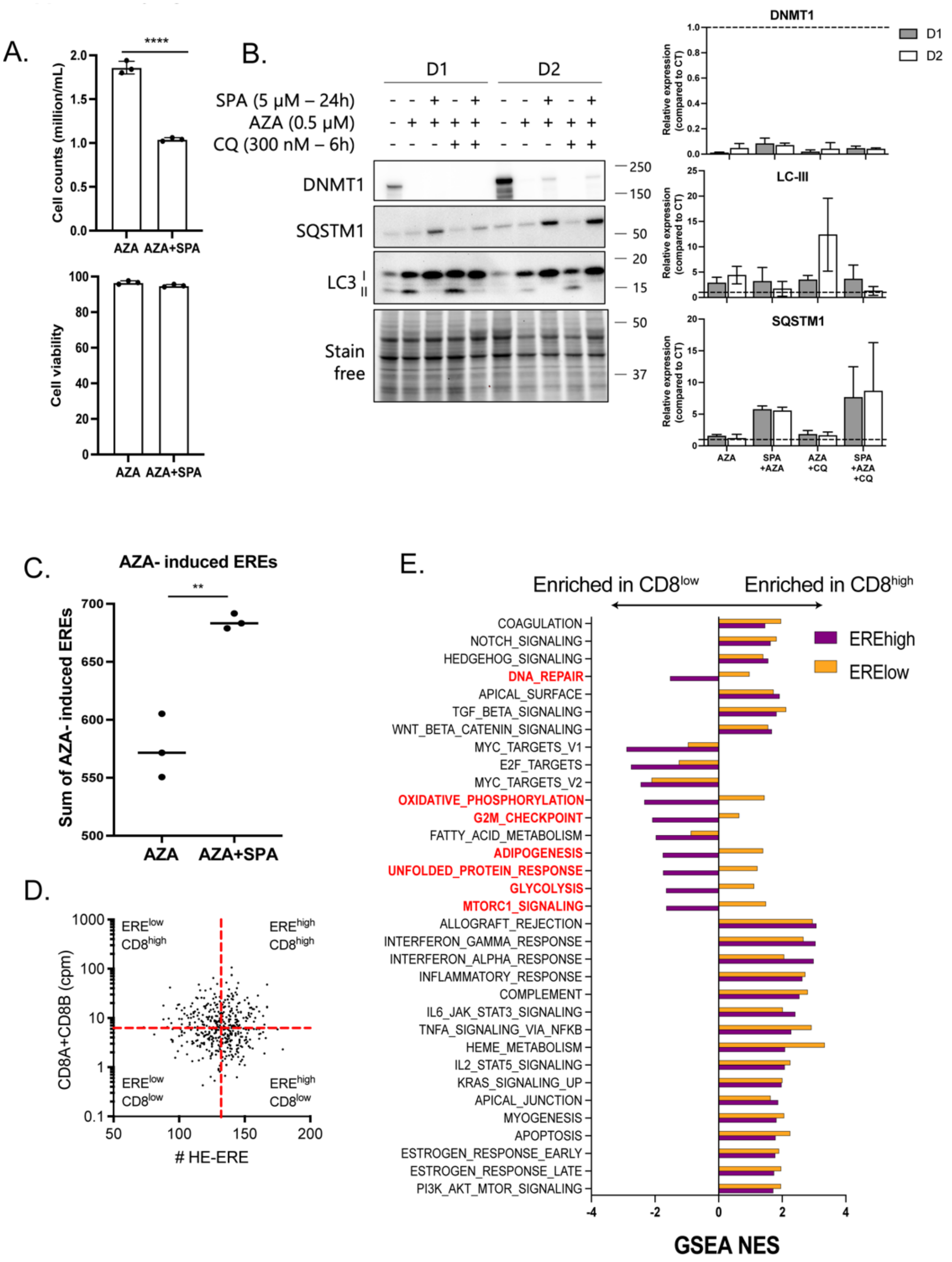

Figure S11. Autophagy inhibition is additive with AZA and increases AML immunogenicity
A, Cell counts and viability of THP-1 cells treated with AZA alone or AZA and Spautin-1, monitored with 7-AAD via flow cytometry (three independent experiments; unpaired t-test; **** p< 0.0001) **B,** Western blot analysis of DNMT1, SQSTM1, and LC3-II protein levels in THP-1 cells after treatment with AZA alone or AZA and Spautin-1 combination (representative blot of three independent experiments) **C,** Quantification of AZA-induced EREs from RNAseq results from THP-1 cells treated with AZA alone or AZA and Spautin-1 combination (unpaired t-test; **

p< 0.01). AZA and Spautin-1 co-treatment experiments refer to with Spautin-1 (5 μM) or DMSO (0.1%) for 24 hours followed by 0.5 μM AZA for three days, followed by 48 hours discontinuation. **D**, Scatterplots of Leucegene AML patients based on their RNA expression of CD8A+CD8B (cpm) vs. their count of highly expressed AZA-induced EREs (HE-EREs: # of AZA-induced EREs whose expression is above their median expression across all patients). **E**, Bar plots indicating normalized enrichment scores for hallmark gene sets between $CD8^{high}$ vs. $CD8^{low}$ Leucegene patients within ERE^{high} and ERE^{low} groups (defined in panel D).

MATERIALS AND METHODS

Cell culture

THP-1, OCI-AML-3, MOLM-13, and SKM-1 cell lines were freshly purchased from the Deutsche Sammlung von Mikroorganismen und Zellkulturen (DSMZ) for the current study. THP-1, MOLM-13, and SKM-1 cells were maintained in RPMI 1640 (Gibco, NY-US, 11875-093) containing L-glutamine and supplemented with 10% heat-inactivated fetal bovine serum (FBS, Gibco 12483) and 1% penicillin-streptomycin (10,000 U/mL, Gibco 15140-122). OCI-AML-3 cells were maintained in MEM alpha (Gibco, NY-US, 12571063) containing L-glutamine and nucleotides supplemented with 10% heat-inactivated fetal bovine serum (FBS, Gibco 12483) and 1% penicillin-streptomycin (10,000 U/mL, Gibco 15140-122).

Cell line treatments

For AZA treatments, cell lines were treated daily with 0.25 μM or 0.5 μM of AZA (Sigma Aldrich A2385) for 72h, followed by removal of the drug (replacement of the medium) at time points as indicated in the results section. Levels of genomic 5-methylcytosine after AZA treatment were measured by ELISA with the MethylFlash Global DNA Methylation Kit (Epigentex, P-1030). For DAC treatments, THP-1 cells were treated with 30nM DAC every 24h for 72h, followed by removal of the drug (replacement of the medium), and collection of the cells 24h later. DMSO was added to controls to match the concentration in DAC-treated cells and the concentration was <0.01%. For Spautin-1 dose responses, cell lines were treated with Spautin-1 (Millipore Sigma SML0440) or 0.1% DMSO control for four days, and Spautin-1 or DMSO was replenished when fresh media was added. For co-treatment experiments monitored by confocal microscopy, cells were treated with AZA and Spautin-1 or rapamycin (a kind gift from Guy Sauvageau's lab). THP-1 cells were treated for four days with either Spautin-1 (5 μM), rapamycin (0.5 μM), or 0.1% DMSO in the presence of 0.5 μM of AZA. AZA was added daily for 72h, followed by discontinuation for 24h. Optimized co-treatment to ensure cell proliferation while successfully inhibiting autophagy and DNMT1 was used for RNAseq and MS experiments. This included treatment of cells with Spautin-1 (5 μM) or DMSO (0.1%) for 24 hours followed by AZA (0.5 μM) every 24h for 72h, followed by removal of the drug (replacement of the medium) and collection 48h later.

Flow cytometry

Cells (~1×10^5 cells/sample) were collected and washed 1X with PBS (Sigma P3813) before fixation/permeabilization with either the FOXP3/Transcription Factor Staining Buffer Set (eBioscience) and staining with anti-DNMT1-PE (EPR3522, Abcam) or the Fix Buffer I (Becton Dickinson, BD) followed by Phosflow Perm Buffer III (BD) and staining with anti-H2γX-AF647 (pS139, BD). All staining steps were performed at 4ºC for 30 min in the dark, and cells were pre-incubated with Fc receptor blocking antibody (BD Pharmingen 552930) for 10 min before incubation with antibodies of interest. Data were acquired on a FACS Canto II (BD).

For protein aggregate detection, 1×10^5 cells/sample were washed 3X with PBS and then fixed/permeabilized with the Cytofix/Cytoperm kit (BD) according to the manufacturer's instructions. Cells were then washed 3X with PBS and resuspended in 250 µL of assay buffer (ENZO #51035) supplemented with Proteostat Aggresome detection dye (ENZO #51035) diluted 1:10,000. Cells were analyzed with a FACS Canto II (BD) after 30 min of staining without additional washes.

Autophagy activity was measured using an autophagy assay kit (Abcam, ab139484) according to the manufacturer's protocol. In brief, cells (~2×10^5/sample) were cultured for 24h in various concentrations of AZA, DAC, or rapamycin in the presence of 60 µM of chloroquine (to accumulate autophagic granules and enable their detection). Cells were collected by centrifugation and washed with assay buffer before being resuspended in 250 µL of culture medium containing 5% FBS and mixed with 250 µL of diluted green staining solution. Cells were incubated for 30 min at 37ºC in the dark and washed with assay buffer. Relative autophagy activities were measured using a Cytoflex flow cytometer (Beckman Coulter) or a FACS Canto II flow cytometer (DB).

Immunoproteasome activity

Immunoproteasome activity was assessed on THP-1 or OCI-AML-3 cells (~5×10^5 cells/sample) lysed in 1 mL of lysis buffer (50 mM Tris-HCl, 2 mM DTT, 5 mM $MgCl_2$, 10% (v/v) glycerol, 2 mM ATP, and 0.05% (v/v) digitonin). The assay was performed immediately after lysis with the Immunoproteasome Activity Fluorometric Assay Kit I (Ubiquitin-Proteasome Biotechnologies, TX-US, J4160) according to the manufacturer's instructions. Fluorescence was detected using the TriStar^2 LB 942 microplate reader (Berthold Technologies GmbH & Co.KG).

Transfections and siRNA knock-down

Transfections were performed, according to a protocol adapted from PMID 33556144, on THP-1 cells with the Lipofectamine 3000 kit (Invitrogen, CA, USA). Briefly, 75×10^4 THP-1 cells were transfected with complexes containing 10 nM siRNA (Invitrogen #s4220) or containing 10nM of scramble (Invitrogen #4390843) and 6 µl of Lipofectamine in 150 µl of Opti-MEM medium in 6-well plates. The cells were incubated with siRNA–lipid complexes for 24h, after which the cells were washed and cultured for an additional 24h in complete medium before performing protein aggregates detection.

Real-time PCR

Quantitative real-time PCR was performed for candidate ERE and dsRNA-induced interferon genes using validated Universal ProbeLibrary assays (Roche) on the Viia7 Real-time PCR system (Applied Biosystems). Relative target mRNA levels were normalized to GAPDH and ACTIN and analyzed using Expression Suite software v1.1 (ThermoFisher).

Immunofluorescence experiments

THP-1 cells were attached on chambered slides (iBidi 80826) with poly-L-lysine and fixed using ice-cold methanol for 15 min at -20ºC, washed three times with PBS, and incubated with saturation

buffer (5% BSA-PBS) for 1h. Primary antibody was added (1:200, anti-dsRNA, clone J2, SCICONS) and incubated overnight at 4ºC. Cells were washed three times for 15 minutes with PBS on a shaker, followed by incubation with secondary antibodies (1:2000 goat anti-mouse IgG AlexaFluor 594 Invitrogen A-11020) at room temperature for 1h and washed three times for 10 minutes with PBS. Next, cells were incubated with DAPI containing PBS, and slides were stored at 4ºC in the dark for at least three days before confocal analyses. Confocal analyses were performed with a Zeiss LSM700 confocal microscope, and images were quantified using EBImage package on R. Transfected cell lines with Poly(I:C) were used as positive controls. No unspecific staining was observed with secondary antibodies alone.

Western Blot Experiments

Proteins were extracted from THP-1 cell line using RIPA lysis buffer (50 nM Tris-HCl, 150 mM NaCl, 0.1% SDS, 0.5% deoxycholate and 1% NP-40) containing phosphatase and protease inhibitors (Thermo Fisher Scientific, PIA32961) for 20 min at 4°C and stocked at −80°C until their centrifugation at 16,000 × g for 10 min before western blotting. Protein concentration was quantified using Bradford assay (BioBasic, BDE641). Twenty to thirty micrograms of protein were loaded in precast 4-15% gradient Stain-Free gels (4–15% Mini-PROTEAN™ TGX Stain-Free™ Protein Gels, BioRad, 4568086) and transferred with Trans-Blot Turbo Mini 0.2 µm nitrocellulose membranes (Bio-Rad, 1704158). Membranes were blocked 45 min at room temperature (RT) with 5% milk in PBS-Tween and incubated overnight at 4°C with primary antibody in BSA 1%. The day after, membranes were incubated for 1h at RT with appropriate secondary antibody for 1h. Protein signals was detected using ChemiDoc MP Imaging System (Bio-Rad). Densitometric analysis was performed using the ImageLab software (Bio-Rad) and Stain-Free lane was used for protein expression normalization.

Library preparation and RNA sequencing

Total RNA was isolated using TRIzol (Thermo Scientific) followed by RNeasy purification (Qiagen). RNA was quantified using Qubit (Thermo Scientific), and quality was assessed with the 2100 Bioanalyzer (Agilent Technologies). Transcriptome libraries were generated using the KAPA RNA HyperPrep (Roche) using a poly-A selection (Thermo Scientific). Sequencing was performed on the Illumina NextSeq500, obtaining around 120M paired-end reads per sample (60M clusters) for AML cell lines.

Gene expression analyses

All transcript expression (canonical genes and EREs) quantifications were performed with kallisto v0.43.0 (1) with default parameters. Kallisto's transcript-level count estimates were converted into gene-level counts using the R package tximport. EdgeR was used to normalize counts using the TMM algorithm and output count-per-million (cpm) values. Differential gene expression analyses were conducted in R3.6.1, as reported previously (2). In brief, raw read counts were converted to

cpm, normalized relative to library size, and lowly expressed genes were filtered by keeping genes with cpm >1 in at least two samples using edgeR 3.26.8 (3) and limma 3.40.6 (4). Subsequently, voom transformations and linear modeling using limma's lmfit were performed. Moderated t-statistics were then computed with eBayes. Genes with false-discovery rates ≤0.05 and $-1 \geq \log_2(FC) \geq 1$ were considered significantly differentially expressed. For differential gene expression analyses performed on AZA-treated cell lines, a unique paired analysis comparing AZA-treated vs. control cells was performed.

Gene ontology and biological pathway annotations were performed with DAVID v6.8 (https://david.ncifcrf.gov). Functional annotations with a p-value <0.05 were considered significant. Gene set enrichment analysis (GSEA) was performed with the fgsea package in R (5) on a pre-ranked gene list generated by ranking expressed genes obtained from limma-voom on moderated t-statistics. Gene sets were obtained either from the HALLMARK or REACTOME matrix, downloaded from the MSigDB database. Single-sample gene set enrichment analyses (ssGSEA) for all REACTOME gene sets were performed using the GSVA package (PMID 23323831) in R on cpm- and TMM-normalized gene set expression data obtained from Kallisto and normalized with limma. Correlations between ssGSEA enrichment values and the sum of AZA-induced ERE expression (in cpm) was performed in R with the cor.test function in R and obtained p-values were corrected with FDR method of the p.adjust function.

Database generation for mass spectrometry identifications

To build databases to analyse MAPs originating from any region of the genome (canonical exons, introns, EREs, ncRNAs, intergenic regions, etc.) and including MAPs deriving from mutations present in the genome of the analyzed cell line, we adopted an alignment-free proteogenomic approach. We built two personalized, non-overlapping proteomes, canonical and non-canonical, and concatenated them to perform MS identifications. All scripts and usage instructions for the pipeline can be found on Zenodo (doi 10.5281/zenodo.7096388).

Personalized canonical proteomes

RNA-Seq reads were trimmed using Trimmomatic v0.35 and aligned to GRCh38.88 using STAR v2.5.1b (6) running with default parameters except for --alignSJoverhangMin, --alignMatesGapMax, --alignIntronMax, --quantMode and --alignSJstitchMismatchNmax parameters for which default values were replaced by 10, 200,000, 200,000, TranscriptomeSAM and "5 -1 5 5", respectively, to generate bam files. Single-base mutations with a minimum alternate count setting of 5 were identified using freeBayes 1.0.2-16-gd466dde (7). Transcript expression was quantified in transcripts per million (tpm) with kallisto v0.43.0 with default parameters. Finally, we used pyGeno (8) to insert high-quality sample-specific single-base mutations (freeBayes quality >20) into the reference exome and export sample-specific sequences of known proteins generated by expressed transcripts (tpm >0) to generate fasta files of personalized canonical proteomes.

Personalized non-canonical proteomes

Step 1. We built consensus genomes and transcriptomes (including only genomic regions covered by RNA-seq reads and single-nucleotide polymorphisms as ambiguous nucleotides) from STAR-generated bam files of each sample (per replicate per cell line). This was performed with the reference genome and transcriptome as input of the samtools (9) and bcftools suites (10): 'samtools mpileup -C50 -uf reference.fasta sample.bam | bcftools call -c | vcfutils.pl vcf2fq | gzip >> consensus.fastq.gz'. The consensus genomes and transcriptomes were then chopped into k-mers (of 24, 27, 30, or 33 nucleotide lengths, corresponding to the length of MAPs: 8–11 amino acids) with a homemade python script, and k-mers containing consensus nucleotides (R|Y|M|K|W|S|B|D|H|V|N) were disambiguated (A|T|C|G) using a homemade python script. For the ERE-focused analyses shown in Figures 5 and 6, the consensus genome was first filtered to keep only ERE-overlapping regions (with bedtools getfasta and a bed file of ERE regions obtained from Repeatmasker) before chopping it into k-mers. These k-mers were then reverse-complemented, and all k-mers (non-ambiguous and disambiguated, originals and reverse-complemented) were assembled in a single database generated with Jellyfish v2.2.3 (11).

Step 2. The fastq files of each sample (per replicate per cell line) were used to generate k-mer libraries of either 24, 27, 30, or 33 nucleotides in length containing k-mers present at least twice per sample. This was performed with Jellyfish: 'jellyfish count -L 2 -m <length> -F 2 -s 1G -o sample.jf' on trimmed forward and reverse-complemented (with the fastx_reverse_complement function of the FASTX-Toolkit v0.0.14) reverse fastq files. Next, the k-mer databases were combined into single databases per cell line (four databases were obtained eventually, one per MAP length) by keeping only those k-mers with three occurrences in three different samples (out of six samples: three controls and three AZA-treated). This was performed with a script ('joinCounts') obtained from the DE-kupl pipeline (12): 'joinCounts -r 3 -a 3 <fastq files>'. This allowed us to retain k-mers that most likely generate MAPs (since high RNA expression is a robust predictor of MAP generation (13,14)).

Step 3. The k-mers generated in step 2 were queried in the k-mer databases generated in step 1. This allowed us to discard consensus artifacts such as exon-intron junctions, wrong SNP calling, false intron coverage and to filter k-mers on their minimum occurrence and inter-sample sharing. The query was performed with the 'jellyfish query -i' command.

Step 4. The personalized canonical proteomes were chopped into peptide k-mers (8, 9, 10, or 11 amino acids) using a homemade python script.

Step 5. The resulting k-mers from step 3 were translated into their peptide sequence with a homemade python script. Peptide k-mers containing stops were removed (with awk), and peptide k-mers generated in step 4 were removed from this list to prevent overlaps between the canonical and non-canonical proteome.

Step 6. The non-canonical peptides were tested for their capacity to bind HLA alleles of their respective cell line (determined with Optitype (15)) with either MHC flurry 1.4.0 (16) or netMHCpan 4.0 (17) for alleles not supported by MHC flurry. Predictions were made with the

epitopepredict module (18) to handle MHC flurry and NetMHCpan. Peptides with a percentile rank ≤2% were kept for further processing.

Step 7. Since leucine and isoleucine variants are not distinguishable by standard MS approaches, we inspected the list of non-canonical peptides and discarded those for which an existing variant (MHC binder as well) was flagged as canonical. Next, short peptides with sequences completely included in the sequence of longer peptides were discarded (awk) from the list, and peptide sequences were used to generate a fasta file, eventually concatenated with the personalized canonical proteome to generate the final MS databases.

MHC-I peptide isolation by immunoprecipitation

W6/32 antibodies (BioXcell) were incubated in PBS for 60 min at room temperature with PureProteome protein A magnetic beads (Millipore) at a ratio of 1 mg of antibody per 1 mL of slurry. Antibodies were covalently cross-linked to magnetic beads using dimethylpimelidate as described (19). The beads were stored at 4ºC in PBS (pH 7.2) and 0.02% NaN_3. Frozen cell pellets (118–135×10^6 cells/pellet) were thawed and resuspended in 0.4 mL PBS (pH 7.2) and solubilized with 1 mL of detergent buffer containing PBS (pH 7.2) and 1% (w/v) CHAPS (Sigma) supplemented with a protease inhibitor cocktail (Sigma). Cell pellets were incubated for 60 min with tumbling at 4ºC and then spun at 16,600g for 20 min at 4ºC. Supernatants were transferred into new tubes containing 1 mg of W6/32 antibody covalently-cross-linked protein A magnetic beads per sample and incubated with tumbling for 20h at 4ºC. Samples were placed on a magnet to recover bound MHC-I complexes to magnetic beads. Magnetic beads were first washed with 8× 1 mL PBS, then with 1× 1 mL of 0.1X PBS, and finally with 1× 1 mL of H_2O. MHC-I complexes were eluted from the magnetic beads by acidic treatment using 0.2% formic acid (FA). To remove residual magnetic beads, eluates were transferred into 2.0 mL Costar mL Spin-X centrifuge tube filters (0.45 mm, Corning) and spun for 5 minutes at 855g. Filtrates containing peptides were separated from MHC-I subunits (HLA molecules and β-2 macroglobulin) using homemade stage tips packed with two 1 mm diameter octadecyl (C-18) solid phase extraction disks (EMPORE). Stage tips were pre-washed with methanol, then with 80% acetonitrile (ACN) in 0.1% trifluoroacetic acid (TFA), followed by 0.1% TFA, and finally with 1% TFA. Samples were loaded onto stage tips and washed with 1% TFA, followed by 0.1% TFA. Peptides were eluted with 30% ACN in 0.1% TFA, dried using vacuum centrifugation, and then stored at -20ºC until MS analysis.

TMT labeling

MHC-I peptide extracts were reconstituted in 200 µL of 200 mM HEPES buffer (pH 8.2). TMT0-126 reagents or TMT6-plex (Thermo Fisher Scientific) were dissolved in 40 µL of anhydrous ACN (Sigma-Aldrich), and 5 µL of 0.02 mg/ µL was added to the peptides. The solutions were gently mixed and incubated for 90 min without agitation at room temperature before the reactions were quenched by hydroxylamine (Thermo Fisher Scientific). Samples were desalted on homemade C18 stage tips and dried down.

Mass spectrometry analyses

Dried peptide extracts from AZA-treated cells (pertaining to figure 2F) were resuspended in 4% FA (EMD Millipore) and loaded on a custom C18 analytical column (20 cm × 150 mm i.d. packed with C18 Jupiter Phenomenex) with a 106-min gradient from 0% to 30% ACN (0.2% FA) and a 600 nL/min flow rate on an EasynLC II system. Samples were analyzed with an Exploris mass spectrometer (Thermo Fisher Scientific) in positive ion mode with the source at 2.8 kV. Each full MS spectrum, acquired with 240,000 resolution, was followed by MS/MS spectra, where the most abundant multiply charged ions were selected for MS/MS sequencing with a resolution of 30,000, 100% normalized automatic gain control, injection time of 700 ms, and collisional energy of 36%. For Decitabine and AZA and Spautin-1 combination experiments, dried peptide extracts were analyzed directly without any TMT labeling. They were resuspended in 4%FA (EMD Millipore) and loaded on an Aurora Ultimate analytical column (25 cm x 75 um i.d., IonOpticks) with a 106 min gradient from 7% to 38% ACN (0.2% FA) with a 300 nL/min flow rate on a Vanquish Neo UHPLC system. Samples were analyzed with an Orbitrap Ascend Tribrid mass spectrometer (Thermo Fisher Scientific) in positive ion mode with the source at 3 kV. Each full MS spectrum, acquired with 120,000 resolution was followed by MS/MS spectra, where the most abundant multiply charged ions were selected for MS/MS sequencing with a resolution of 30,000, 100% normalized automatic gain control, injection time of 59 ms, and collisional energy of 27%.

Identification of MAPs and differential MAP analyses

Liquid chromatography (LC)-MS/MS (LC-MS/MS) data were searched against respective cell line-specific databases using PeaksXPro. For peptide identification, no enzyme was selected, and tolerance was set at 10 ppm and 0.01 Da for precursor and fragment ions, respectively. The occurrences of oxidation (M) and deamidation (NQ) were set as variable modifications. Following peptide identification, we used a modified target decoy approach built-in PEAKS and applied a sample-specific threshold on the PEAKS score to ensure a false discovery rate of 5%, calculated as the ratio between the number of decoy hits and the number of target hits above the score threshold. Binding affinities to the sample's HLA alleles were predicted with NetMHCpan 4.1b (20), and only 8 to 11-amino-acid-long peptides with a rank eluted ligand threshold ≤ 2% were used for further annotation; these filtering steps were performed with MAPDP software (21). Intensities of all modifications for a single peptide were summed, and peptides containing too many missing values were eliminated by keeping peptides quantified in two out of three replicates of at least one condition. Next, VSN normalization was performed, which was the best available normalization method based on analyses with NormalyzerDE (22). Imputation for missing values was performed by Perseus with width of 0.3 and downshift of 1, and MAPs with p-values <0.05 and fold-changes (FC)>2 were considered significantly differentially expressed using limma analysis. MAPs exclusively detected in one condition were defined by having valid values from all three biological replicates in one condition while no values in the other condition.

Biotype attribution to identified MAPs

BamQuery (23) was used to annotate if MAPs derived from protein-coding, EREs, or other non-coding regions. CTAs were annotated using (24). Further, Prosit tool (25) was used to evaluate the correlation between predicted vs. experimental spectra of each ERE-MAPs. ERE-MAPs with Prosit spectral angle value below 0.6 were considered good candidates.

Bioinformatic analyses performed on MAPs

Amino acid compositions, aromaticity, and GRAVY indexes were assessed with the ProtParam module of Biopython. The RNA expression of each MAP was obtained using BamQuery (23). Immunogenicity predictions of MAPs were performed with Repitope (PMID 31057550). Feature computation was performed with the predefined MHCI_Human_MinimumFeatureSet variable and updated (July 12, 2019) FeatureDF_MHCI and FragmentLibrary files provided on the Mendeley repository of the package (https://data.mendeley.com/datasets/sydw5xnxpt/1). HIV MAPs (positive control) were obtained from https://www.hiv.lanl.gov/content/immunology/tables/ctl_summary.html. Predictions were also performed with the web portal of BamQuery (https://bamquery.iric.ca/search). The RNA expression of MAPs in normal tissues of GTEx, in mTECs, or hematopoietic cells (dataset of hematopoietic cells reported in PMID 33740418) were performed with BamQuery, as described previously (PMID 37582761). MAPs having an expression lower than 8.55 rphm (threshold established in PMID 33740418) were defined as TSA.

Quantification and statistical analysis

A log fold-change higher than 1 was defined as the desired size effect. Power calculations were performed with the PROPER package in R and revealed an average marginal power of 0.86 for differential gene expression analysis when using 12 samples per condition. Therefore, four cell lines in triplicate were used as the main dataset.

Unless indicated otherwise, all statistical tests comparing two conditions were performed using the Mann–Whitney U test. All correlations were assessed with the Pearson correlation coefficient. Unless mentioned otherwise, all boxes in boxplots represent the median, 25th, and 75th percentiles, and whiskers extend to the 10th and 90th percentiles. Unless mentioned otherwise, all bar plots represent the average with standard deviation (SD). Plots and statistical tests were mainly performed with GraphPad Prism v9.1.1. For all statistical tests, **** refers to $p< 0.0001$, *** refers to $p< 0.001$, ** refers to $p< 0.01$, and * refers to $p< 0.05$.

Data and code availability

The accession number for the RNA sequencing and expression data reported in this paper is GEO:GSE217572. MS raw data and associated databases are deposited to the ProteomeXchange Consortium via the PRIDE partner repository with the following dataset identifiers: PXD038663 and PXD046853. The code necessary for the generation of MS databases has been deposited on Zenodo https://doi.org/10.5281/zenodo.7096388.

References

1. Bray NL, Pimentel H, Melsted P, Pachter L. Near-optimal probabilistic RNA-seq quantification. Nature Biotechnology **2016**;34:525-7
2. Noronha N, Ehx G, Meunier MC, Laverdure JP, Thériault C, Perreault C. Major multilevel molecular divergence between THP-1 cells from different biorepositories. Int J Cancer **2020**;147:2000-6
3. Robinson MD, McCarthy DJ, Smyth GK. edgeR: a Bioconductor package for differential expression analysis of digital gene expression data. Bioinformatics **2010**;26:139-40
4. Ritchie ME, Phipson B, Wu D, Hu Y, Law CW, Shi W*, et al.* limma powers differential expression analyses for RNA-sequencing and microarray studies. Nucleic Acids Res **2015**;43:e47
5. Sergushichev AA. An algorithm for fast preranked gene set enrichment analysis using cumulative statistic calculation. bioRxiv **2016**:060012
6. Dobin A, Davis CA, Schlesinger F, Drenkow J, Zaleski C, Jha S*, et al.* STAR: ultrafast universal RNA-seq aligner. Bioinformatics **2013**;29:15-21
7. Garrison E, Marth G. Haplotype-based variant detection from short-read sequencing. arXiv: Genomics **2012**
8. Daouda T, Perreault C, Lemieux S. pyGeno: A Python package for precision medicine and proteogenomics. F1000Res **2016**;5:381
9. Li H, Handsaker B, Wysoker A, Fennell T, Ruan J, Homer N*, et al.* The Sequence Alignment/Map format and SAMtools. Bioinformatics **2009**;25:2078-9
10. Li H. A statistical framework for SNP calling, mutation discovery, association mapping and population genetical parameter estimation from sequencing data. Bioinformatics **2011**;27:2987-93
11. Marcais G, Kingsford C. A fast, lock-free approach for efficient parallel counting of occurrences of k-mers. Bioinformatics **2011**;27:764-70
12. Audoux J, Philippe N, Chikhi R, Salson M, Gallopin M, Gabriel M*, et al.* DE-kupl: exhaustive capture of biological variation in RNA-seq data through k-mer decomposition. Genome Biology **2017**;18:243
13. Ehx G, Larouche JD, Durette C, Laverdure JP, Hesnard L, Vincent K*, et al.* Atypical acute myeloid leukemia-specific transcripts generate shared and immunogenic MHC class-I-associated epitopes. Immunity **2021**;54:737-52.e10
14. Pearson H, Daouda T, Granados DP, Durette C, Bonneil E, Courcelles M*, et al.* MHC class I-associated peptides derive from selective regions of the human genome. J Clin Invest **2016**;126:4690-701
15. Szolek A, Schubert B, Mohr C, Sturm M, Feldhahn M, Kohlbacher O. OptiType: precision HLA typing from next-generation sequencing data. Bioinformatics **2014**;30:3310-6
16. O'Donnell TJ, Rubinsteyn A, Laserson U. MHCflurry 2.0: Improved Pan-Allele Prediction of MHC Class I-Presented Peptides by Incorporating Antigen Processing. Cell Syst **2020**;11:42-8.e7
17. Reynisson B, Alvarez B, Paul S, Peters B, Nielsen M. NetMHCpan-4.1 and NetMHCIIpan-4.0: improved predictions of MHC antigen presentation by concurrent motif deconvolution and integration of MS MHC eluted ligand data. Nucleic Acids Res **2020**;48:W449-w54
18. Farrell D. epitopepredict: A tool for integrated MHC binding prediction. bioRxiv **2021**:2021.02.05.429892

19. Lamoliatte F, McManus FP, Maarifi G, Chelbi-Alix MK, Thibault P. Uncovering the SUMOylation and ubiquitylation crosstalk in human cells using sequential peptide immunopurification. Nature communications **2017**;8:1-11
20. Reynisson B, Alvarez B, Paul S, Peters B, Nielsen M. NetMHCpan-4.1 and NetMHCIIpan-4.0: improved predictions of MHC antigen presentation by concurrent motif deconvolution and integration of MS MHC eluted ligand data. Nucleic acids research **2020**;48:W449-W54
21. Courcelles M, Durette C, Daouda T, Laverdure J-P, Vincent K, Lemieux Sb*, et al.* MAPDP: a cloud-based computational platform for immunopeptidomics analyses. Journal of proteome research **2020**;19:1873-81
22. Willforss J, Chawade A, Levander F. NormalyzerDE: online tool for improved normalization of omics expression data and high-sensitivity differential expression analysis. Journal of proteome research **2018**;18:732-40
23. Ruiz Cuevas MV, Hardy M-P, Larouche J-D, Apavaloaei A, Kina E, Vincent K*, et al.* BamQuery: a proteogenomic tool for the genome-wide exploration of the immunopeptidome. bioRxiv **2022**:2022.10.07.510944
24. Almeida LG, Sakabe NJ, Deoliveira AR, Silva MCC, Mundstein AS, Cohen T*, et al.* CTdatabase: a knowledge-base of high-throughput and curated data on cancer-testis antigens. Nucleic acids research **2009**;37:D816-D9
25. Gessulat S, Schmidt T, Zolg DP, Samaras P, Schnatbaum K, Zerweck J*, et al.* Prosit: proteome-wide prediction of peptide tandem mass spectra by deep learning. Nature methods **2019**;16:509-18
